# Pathfinder: Protein folding pathway prediction based on conformational sampling

**DOI:** 10.1371/journal.pcbi.1011438

**Published:** 2023-09-11

**Authors:** Zhaohong Huang, Xinyue Cui, Yuhao Xia, Kailong Zhao, Guijun Zhang

**Affiliations:** College of Information Engineering, Zhejiang University of Technology, Hangzhou, China; University of Maryland School of Pharmacy, UNITED STATES

## Abstract

The study of protein folding mechanism is a challenge in molecular biology, which is of great significance for revealing the movement rules of biological macromolecules, understanding the pathogenic mechanism of folding diseases, and designing protein engineering materials. Based on the hypothesis that the conformational sampling trajectory contain the information of folding pathway, we propose a protein folding pathway prediction algorithm named Pathfinder. Firstly, Pathfinder performs large-scale sampling of the conformational space and clusters the decoys obtained in the sampling. The heterogeneous conformations obtained by clustering are named seed states. Then, a resampling algorithm that is not constrained by the local energy basin is designed to obtain the transition probabilities of seed states. Finally, protein folding pathways are inferred from the maximum transition probabilities of seed states. The proposed Pathfinder is tested on our developed test set (34 proteins). For 11 widely studied proteins, we correctly predicted their folding pathways and specifically analyzed 5 of them. For 13 proteins, we predicted their folding pathways to be further verified by biological experiments. For 6 proteins, we analyzed the reasons for the low prediction accuracy. For the other 4 proteins without biological experiment results, potential folding pathways were predicted to provide new insights into protein folding mechanism. The results reveal that structural analogs may have different folding pathways to express different biological functions, homologous proteins may contain common folding pathways, and α-helices may be more prone to early protein folding than β-strands.

## Author summary

The study of protein folding mechanism is an important part of basic science and has vital significance in many aspects. The key to the study of protein folding mechanism is to capture the conformational changes from the fast protein folding process. Biological experiments are more difficult to obtain protein metastable structures than computational methods. It is computationally expensive to simulate the complete folding pathway of macromolecular proteins by molecular dynamics methods. Here, we design a protein folding pathway prediction method based on conformational sampling to provide new ideas for existing research. This method obtains the structural information of the intermediate state through large-scale sampling and clustering, combines the resampling algorithm to explore the transition probability of the intermediate state, and predicts the protein folding pathway. The results show that we validate the method on five widely studied proteins, and also reveal the folding mechanism of some proteins. And Pathfinder complements the existing protein folding data from the perspective of computational simulation, which needs to be further verified by biological experiments. Finally, Pathfinder predicts unresolved protein folding pathways, providing insights into unknown folding mechanisms.

## Introduction

The splendid computational success of AlphaFold2 [1] and RoseTTAFold [2] in protein structure prediction may have solved the static single domain protein folding problem [3]. The AlphaFold database [4] and recent predictions of more than 200 million protein structures provide reference structure information for nearly every known protein [5]. Although, the greatly improved prediction of protein 3D structure from sequence achieved by the AlphaFold2 has already had a significant impact on biological research [6], but challenges remain [7,8]. Almost all computational methods are unable to predict accurate protein folding pathway [9–11]. This is because protein folding is a dynamic process of exploring the overall energy landscape and locating heterogeneous local energy basins to obtain its functional structure and conformations [12]. The study of folding mechanism is of great significance for the formation of inclusion bodies [13] and for revealing the second genetic code [14]. Many pathological conditions are also fundamentally rooted in the misfolding, aggregation, and accumulation that occurs in protein folding [15], such as Alzheimer’s disease [16], Parkinson’s disease [17], and other diseases. Understanding the folding mechanism can provide important implications for the treatment of these diseases [18], as well as facilitate the design of proteins with unique functional characteristics [19,20], and the exploration of protein allosteric [21]. The conformational heterogeneity of different states in protein folding, such as unfolded state [22], misfolded state [23], intermediate state [24], and transition state [25], is crucial for an accurate understanding of folding mechanisms [26].

There are biological experimental methods to explore protein intermediate states and folding pathways [27–32], such as hydrogen deuterium exchange mass spectrometry [33] and circular dichroism spectrum [34]. However, biological experimental methods are difficult to obtain high-resolution spatial and temporal data on the folding process. This is because the biological process by which proteins fold into their unique native state occurs within seconds to minutes [35], and metastable conformations are more difficult to detect due to their short lifetime and low occupancy [36]. This makes it challenging to explore intermediate states with biological experimental methods.

Computational simulation of protein folding can make up for the deficiency of biological experimental methods, and is an effective way of studying protein folding pathways [37]. Molecular Dynamics Simulation (MD) is one of the methods for computationally simulating protein folding, which can simulate the complete folding process of small molecules [38]. MD combined with Markov models can analyze folding and functional dynamics in long trajectories [39]. Machine learning facilitates protein folding simulations by extracting essential information and sampling of rare events from large simulated datasets [40]. Convolutional neural networks learn continuous conformational representations generated from protein folding simulations to predict biologically relevant transition paths [41]. Integrating biological experimental structural constraints into MD models significantly explored protein dynamics trajectories [42]. The current mainstream method is combining biological experiments and MD to explore protein folding mechanism. However, MD are more applied to the simulation of short trajectories between states, and it is still challenging to simulate the complete folding process [37]. Our recent work (PAthreader) [43] identifies remote homologous structures based on the three-track alignment of distance profiles and structure profiles originated from Protein Data Bank (PDB) [44] and AlphaFold database by deep learning. Based on the recognized homologous templates, PAthreader further explored protein folding pathways by identifying folding intermediates, but it has limitations on proteins that lack remote homologous template information.

Conformational sampling algorithms such as Monte Carlo (MC) can be applied to folding simulations of template-free and larger proteins [45]. The CA-CB side chain model combined with MC kinetics to identify the protein folding pathway and the interaction pairs during the folding process [46,47]. Protein folding pathway can be predicted in minutes or hours by predicting residue contacts using coarse-grained modeling and efficient combinatorial schemes [48]. Moreover, Equilibrium Monte Carlo simulations can also be combined with unfolding simulations at high temperatures to predict the relative rates of different transitions in protein folding pathways [49]. Conformational sampling tends to fall into local energy traps. Recently, we have developed several methods (MMpred [50], SNfold [51]) to compensate for this deficiency. The MMpred aims to explore the complete energy landscape and improve sampling efficiency, which can be beneficially applied to explore heterogeneous conformations. MMpred has localized promising energy basins in parallel on multiple trajectories, combining a greedy search strategy with distance-constrained information to infer the final structure. SNfold overcomes high-energy barriers and avoids resampling of exploration regions to obtain diverse heterogeneous conformations in the energy landscape. These state-of-the-art conformational sampling algorithms are mainly used in protein structure prediction. However, the idea of exploring multiple states can be effectively applied to protein folding pathway prediction.

In this work, we propose a protein folding pathway prediction algorithm (Pathfinder) based on conformational sampling. We obtain the structural information of the seed states through large-scale sampling and explore state transition probabilities through resampling. Pathfinder captures the information (seed states, sampling states and transition probabilities) to predict folding pathways. Pathfinder is tested on our developed dataset (34 proteins). For 11 widely studied proteins, we correctly predicted their folding pathways (Fig A in S1 Text) and specifically analyzed 5 of them, including the B1 domain of protein L and protein G, the two SRC homology 3 domains and the LysM domain. For 13 proteins (Fig B in S1 Text), we predicted their folding pathways, which need to be further verified by biological experiments. For 6 proteins (Fig C in S1 Text), we analyzed the reasons for the low prediction accuracy. For the other 4 proteins without biological experiment results, potential folding pathways were predicted to provide new insights into the protein folding mechanism. Analyzing of the above results, we found some protein folding mechanisms.

## Results

### Evaluation metric

Related studies have shown that the logarithm of experimental protein folding rates depends on the local geometry and topology of the protein’s native state [52]. Contact order is a metric of protein topology complexity and stability, reflecting the relative importance of local and nonlocal contacts to protein structure [53]. Contact order has a statistically significant relationship with protein folding dynamics [54]. Contact order accurately assesses the protein folding pathway, especially those whose folds collapse to stabilize globular shapes. However, contact order may have limitations in special cases as shown in Fig D in S1 Text. Therefore, this work verifies the accuracy of the prediction by comparing with the existing biological experiment results. The contact order is defined as:

$$CO=\frac{1}{L\cdot N^{\text{con}}}\sum^{N^{\text{con}}}\Delta S_{ij}$$ (1)

where *N*^con^ is the number of residues whose distance between them is less than 8*Å*. The *ΔS*_*ij*_ is the sequence separation between residue *i* and *j*, and *L* is the length of the protein. On this basis, the residue contact order of *i* -th *R*_co_(*i*) was designed to assess the local folding completion of intermediate states:

$$R_{\text{co}}\left(i\right)=\sum^{N_{i}^{\text{dis}}}\frac{\Delta S_{ij}}{d_{ij}}$$ (2)

where $N_{i}^{\text{dis}}$ is the number of residues whose distance are less than 20*Å* from *i* -th residue. The *d*_*ij*_ is the distances between residue *i* and *j*. The contact order can evaluate the degree of folding completion through the intermediate state structure information. The residue contact order can capture the folding nucleus information and key residue information during the folding process, as described in Fig E in S1 Text.

### Folding pathway with experimental validation

#### The GB1 and LB1

**Fig 1:**
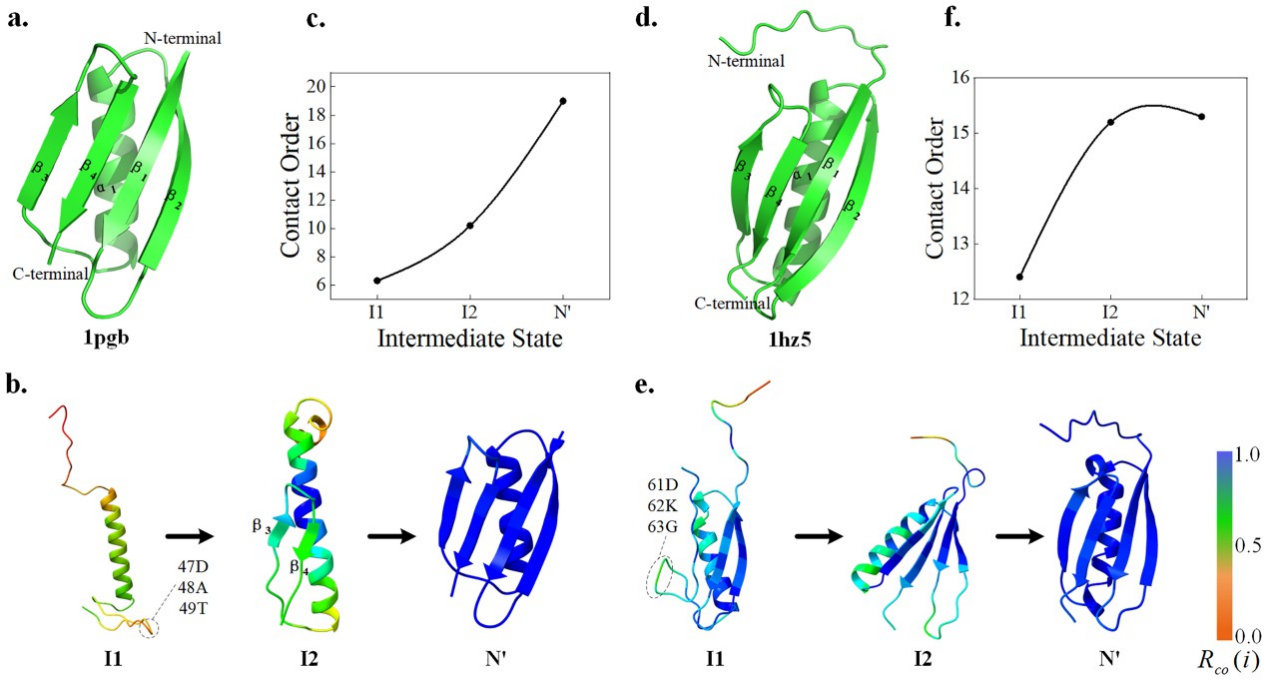
Folding pathway of GB1 (PDB ID:1pgb) and LB1 (PDB ID:1hz5). **(a)** and **(d)** are the native structure of GB1 and LB1. **(b)** and **(e)** are the folding pathway of GB1 and LB1 including intermediate states. **(c)** and **(f)** are the contact orders of the intermediate states. Residue contact order values are normalized and represented on the structure as color.

The IgG-binding B1 domain of protein G (GB1, Fig 1a) and IgG-binding B1 domain of protein L (LB1, Fig 1d) are often used as model proteins for folding mechanism studies. GB1 has a wide range of biomedical uses and studies of protein folding and stability [55], and extensive experimental results and computational simulations exploring the complete folding pathway [46]. Both GB1 and LB1 contain an α-helix and four β-strands, but their sequence similarity is low and their folding pathways differ. Related studies have shown that the hairpin structure (folding nucleus) plays a crucial role in global folding [46].

The predicted protein folding pathways are shown in Fig 1b and 1e. The result shows that GB1 first forms an α-helix, meanwhile the β-turns (47D, 48A, 49T) at the C-terminus has started to form, which may be a sign that I2 of GB1 (called G_I2_) start to form. Then the β_3_ and β_4_ formed as shown in G_I2_. In addition, the β_1_ and β_2_ of G_I2_ is represented in helical, which is different from the native structure in Fig 1a. This is because the unstable structure in the intermediate state usually exists as a disordered region, and it may be replaced by secondary structures such as helices and loops in fragment assembly. Finally, a hairpin structure composed of β_1_ and β_2_ is formed at the N-terminus (as shown in N’). Our GB1 predictions are complete consistent with biological experiments [56–58] and contain structural information for the folding stage. As shown in Fig A in S1 Text, the folding pathway of GB1 can be clearly observed through the residue contact orders in the intermediate state. Therefore, after normalizing the residue contact orders, the color of the scale in the lower right corner of Fig 1 shows the folding degree of the residues.

As shown in 1f, the I1 of LB1(called L_I1_) suggest that the β_1_ and β_2_ hairpin structures at the N-terminal are formed earlier than the β_3_ and β_4_ structures, which was different from the folding mechanism of GB1. After β_3_ and β_4_ folding, the loop of LB1 folds to stabilize. Similarly, C-terminal β turns (61D, 62K, 63G) and β_4_ start to form in the L_I1_. The predicted results of LB1 are almost consistent with the biological experiments [59], but the order of helix and N-terminal hairpin structure formation is missing. By analyzing the conformational structure of the sampling process (Fig F in S1 Text), we found that the sampling occupancy rate of these two structures is low, and the clustering structure is mainly the process of β_3_ and β_4_ and loop. This is because the fragment assembly method completes both the helix and sheet folding early in conformational sampling.

As shown in Fig 1c and 1f, the contact order of their intermediate states showed an upward trend, reflecting the degree of globular protein folding. The folding of L_I2_ to N’ is mainly affected by loop adjustment, and the degree of folding is much lower than that of β-strand formation from the I1 of LB1 to L_I2_. In addition, related studies have shown that the formation order of the hairpin structure is crucial to the folding rate and stability [57]. The above results suggest that structural analogs may have different folding pathways and provide material for protein design.

Inspired by this difference in folding mechanism, the GB1 mutant NuG2 (PDB ID: 1mi0) was designed [59]. We also predicted the folding pathway of the NuG2 as shown in Fig A in S1 Text. The results show that NuG2 not only has the folding pathway of LB1 protein, but also may have the folding pathway of the original GB1 protein as shown in Fig G in S1 Text.

### SRC Homology 3 Domain

The study on the folding of SRC Homology 3 Domain provides extremely valuable information for the molecular mechanism of amyloid formation and the cytotoxicity of protein aggregates, which is of great significance for better understanding the pathological process and exploring the possibility of future treatment [60]. Extensive experimental and theoretical studies explored the natively stable intermediate states and complete folding pathways of the SH3 protein, and found that the unfolded state of β_5_ may be responsible for misfolding [61].

**Fig 2:**
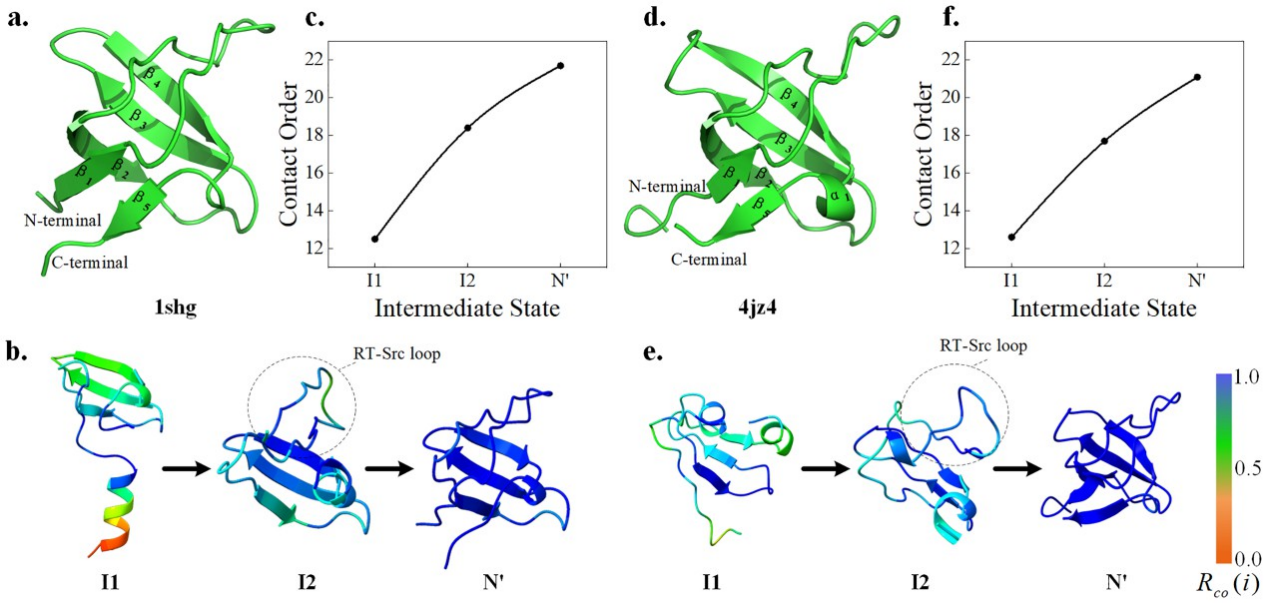
Folding pathways of eSH3 (PDB ID:1shg) and cSH3 (PDB ID:4jz4). **(a)** and **(d)** respectively their native structures. **(b)** and **(e)** are folding pathway including the intermediate states. **(c)** and **(f)** are contact order of intermediate states.

Here, we predicted the folding pathway of SH3 from Escherichia coli (eSH3, Fig 2a) and chicken c-Src-SH3 domain (cSH3, Fig 2d). Interestingly, the folding pathways and the contact order of intermediate states are almost identical for the two proteins. First, the I1 in Fig 2b and 2e show that a folded nucleus consisting of β_2_, β_3_, and β_4_ forms. Then, both loops marked in I2 of Fig 2b and 2e is called RT-Src loop, which is gradually formed. Finally, the folding nucleus is used as the support point to drive the formation of β_1_ and β_5_ at the N-terminal and C-terminal of the protein to complete the folding. The predicted results are completely consistent with the biological experimental results [62], which verifies the effectiveness of Pathfinder. Although there are differences in the intermediate state structures of the two SH3 proteins, the order of key local structures is highly consistent. Furthermore, we respectively predict the folding pathways of three homologous proteins of Escherichia coli SH3 protein mutant (PDB ID: 1srl), Caenorhabditis elegans SH3 (PDB ID: 1b07) and human Fyn SH3 (PDB ID: 5zau) in Fig A in S1 Text. In these SH3, the formation of folded nucleus composed of β_2_, β_3_, and β4 can be observed, and the RT-Src loop usually only forms a rough outline. Therefore, it may be the instability of the RT-Src loop leads to insufficient constraints on β_5_ during SH3 folding, leading to misfolding. The result also show that these homologous proteins may share the same folding pathway.

#### LysM domain

The lysin domain (LysM) is a ubiquitous and versatile peptidoglycan-binding module found in bacterial proteins [63]. Because of the simple structure and important biological significance of this protein, a large number of studies have analyzed the folding transition state and folding pathway of this protein [64–66]. The protein consists of 48 residues with a secondary structure arrangement of βααβ and a highly robust folding pathway.

**Fig 3:**
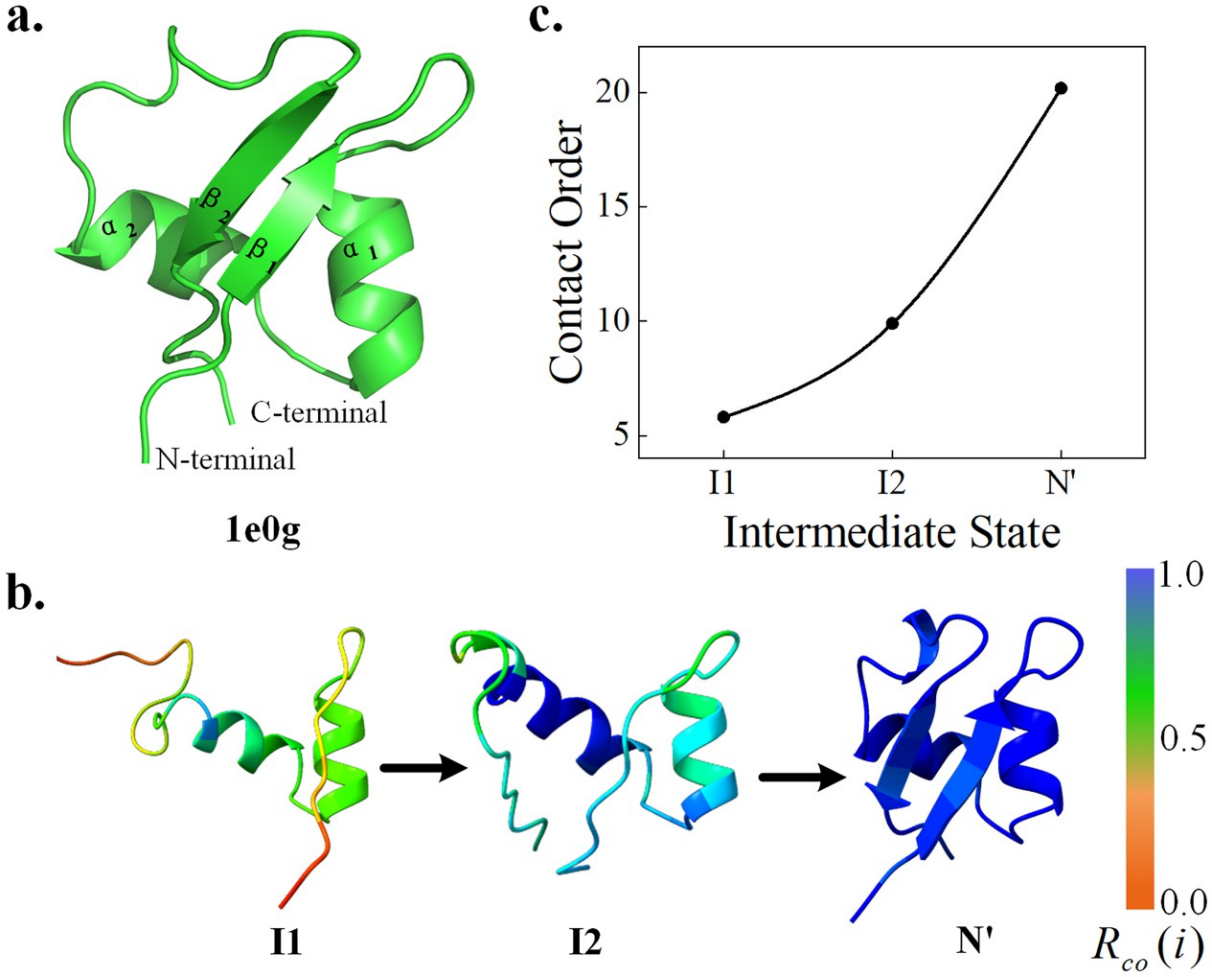
Folding pathway of LysM (PDB ID: 1e0g). **(a)** is the native structure of the LysM protein. **(b)** is the contact order of the intermediate states. **(c)** is the folding pathway including the intermediate states and their residue contact order.

The folding pathway predicted by Pathfinder is shown in Fig 3b, which includes the intermediate state of only α-helix formation and the process of β-strand formation. The folding pathway of LysM is relatively clear, and the folding degree of residues can be clearly analyzed through the residue contact order. The two α-helices in the middle of I1 form a folding nucleus. The inward extrusion of the two stable α-helices then drives the two ends to fold (intermediate state 2). Finally, two β-strands formation of LysM completes the folding. The result is highly consistent with biological experiments [67].

By comparing the intermediate state data or folding pathway information collected in relevant literature with the predicted results by Pathfinder, we verified the folding pathways of 11 proteins. The above is the specific analysis of 5 proteins, and the folding pathways of the remaining 6 proteins are shown in Fig A in S1 Text.

### Protein folding pathways to be validated

Because it is difficult to obtain the intermediate state, biological experiments usually study the folding mechanism by analyzing the key residues in the folding process. Pathfinder predicts protein heterogeneous conformation and folding pathways by sequence and native state. This may provide new ideas for the study of folding mechanism. The 13 proteins in Fig B in S1 Text require further validation because of the lack of intermediate state data. In addition, we found that for proteins with both α-helices and β-strands, the initial intermediate state often contains helical structures. Furthermore, α-helical structures are generally believed to be more stable due to having more hydrogen bonds than β-strands. Therefore, we thought that α-helices might generally be easier to form early in folding.

### Folding pathway without experimental validation

**Fig 4:**
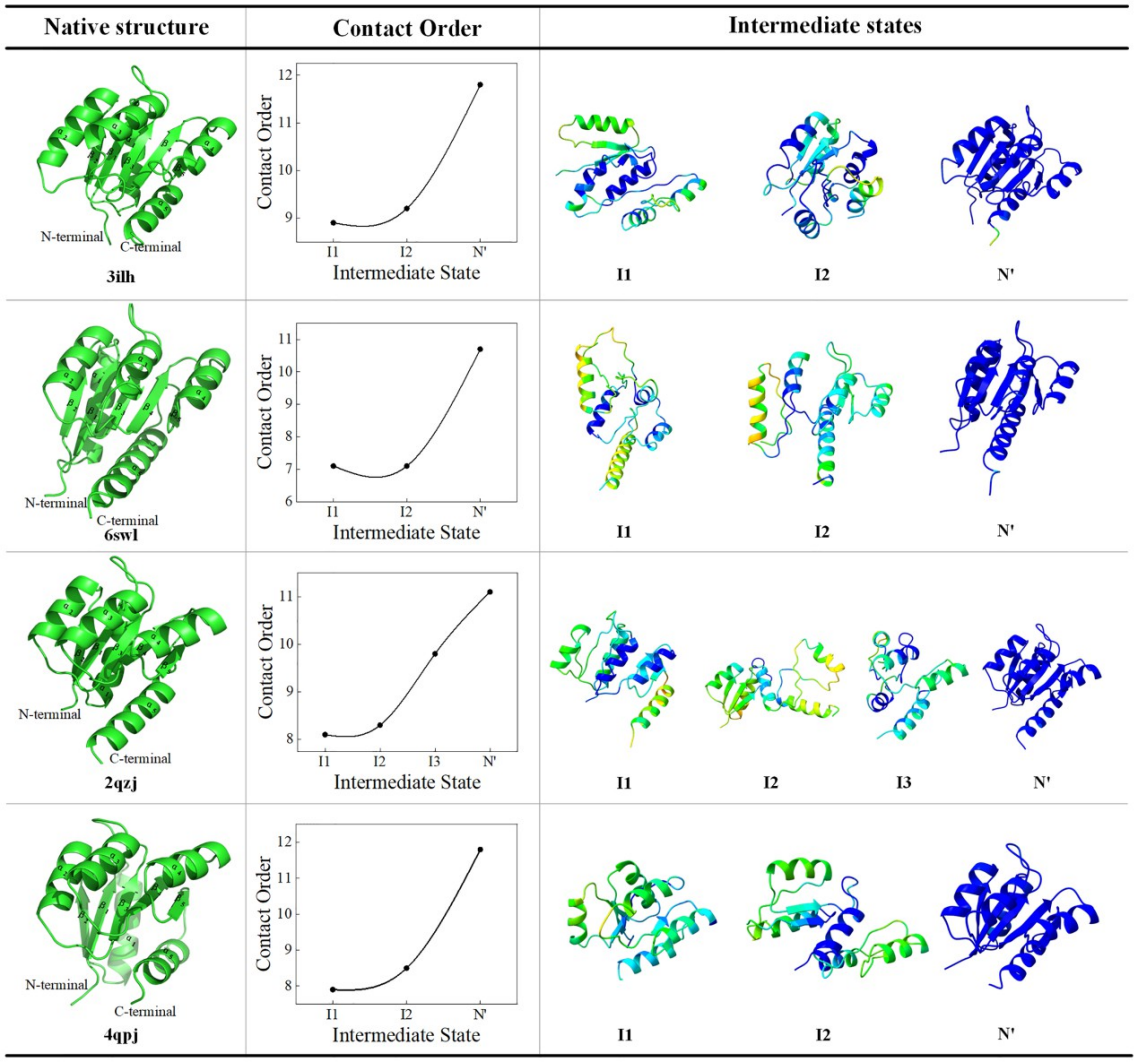
Folding pathways of four unexperimented proteins. Folding pathways include intermediate state structure information and normalized residue contact order. The color annotation of residue contact order is consistent with the intermediate state in Fig 1.

Response regulator proteins utilize distinct molecular surfaces in inactive and active conformations for various regulatory intramolecular and intermolecular protein interactions [68]. Molecular dynamics simulations complement structural studies of conformational changes under receptive domain switch function [69]. However, access to the heterogeneous conformation required for MD is not easy. Pathfinder predicted the folding pathways of four related proteins (as show in Fig 4), including a two-component response regulator from Cytophaga hutchinsonii (PDB ID: 3ilh), a response regulator from Geobacillus stearothermophilus (PDB ID: 6swl), a two-component response regulator from Clostridium difficile (PDB ID: 2qzj), and a phosphotransferase in complex with a receiver domain (PDB ID: 4qpj).

Their contact orders all increase exponentially, indicating that the intermediate conformational structure occurs more in the early stage of folding. In the early stage of folding, these proteins all form multiple α-helices, further illustrating that the helical structure may be formed earlier than the β-strand. During the subsequent folding pathway, internal β-strands are formed step by step. By comparing the intermediate states of these proteins, we found that several α-helices and β-strands often form a super-secondary structure or folded nucleus. It is the mutual extrusion and collapse of these folded nuclei that stabilize the protein fold. Pathfinder can predict the protein folding pathway by sequence and native to study the protein folding mechanism of the unresolved native conformation.

## Discussion

We apply efficient sampling algorithms to explore intermediate states and develop a protein folding pathway prediction algorithm based on conformational sampling. Pathfinder captures information between intermediate states to predict protein folding pathways. The results show that Pathfinder can extract the commonality from the folding pathways of multiple proteins, and discover the folding mechanisms of some proteins. For example, structural analogs may have different folding pathways to express different biological functions and provide insights for protein design. The proteins of the SH3 family may have the same folding pathway, and the instability of the loop region leads to insufficient force on the local structure, resulting in misfolding. During folding, α-helices may form earlier than β-strands because of the influence of hydrogen bonds. Further, we explore the predictive preference of Pathfinder. We found that Pathfinder more easily predicted proteins containing both β-strand or α-helix structures as shown in result. For proteins containing only α-helix it may be biased due to the easier assembly of helices by fragment assembly (Fig H in S1 Text).

As shown in Fig I in S1 Text, we performed a basic protein conformation sampling procedure on the 1e0m protein and the 1opa protein to analyze their correctness of energy force fields. The results show a situation where 1e0m protein energy cannot be reduced. This also shows that the appropriateness of the energy force field affects the accuracy of protein folding pathway prediction. Moreover, we analyzed the folding pathways of 6 proteins whose prediction accuracy was insufficient as shown in Fig J in S1 Text. By evaluating the energy of the intermediate state, we found that the energy of the intermediate state tended to decrease under the *ref2015* energy force field. However, the evaluation of the contact order showed that the degree of folding did not show a clustering trend. Therefore, the inaccuracy of the energy force field leads to the flawed prediction of the folding pathway. Since Pathfinder is based on the simulation of protein folding guided by the Rosetta force field. Energy force fields trained with deep learning may be applicable to folding pathway prediction for more proteins.

Compared with traditional biological experiments, Pathfinder obtains approximate protein intermediate state structures and, at the same time, predicts the order in which these intermediate states appear, enriching protein folding data. For molecular dynamics simulations, if the protein sequence is too long, the vast calculation parameters will inevitably limit the simulation of the folding process. The method can be combined with molecular dynamics simulations to provide new insights into methods for computationally simulating protein folding. At the same time, Pathfinder can analyze family proteins or protein collections under different classifications, and explore protein folding mechanisms from a broader perspective.

## Materials and methods

**Fig 5:**
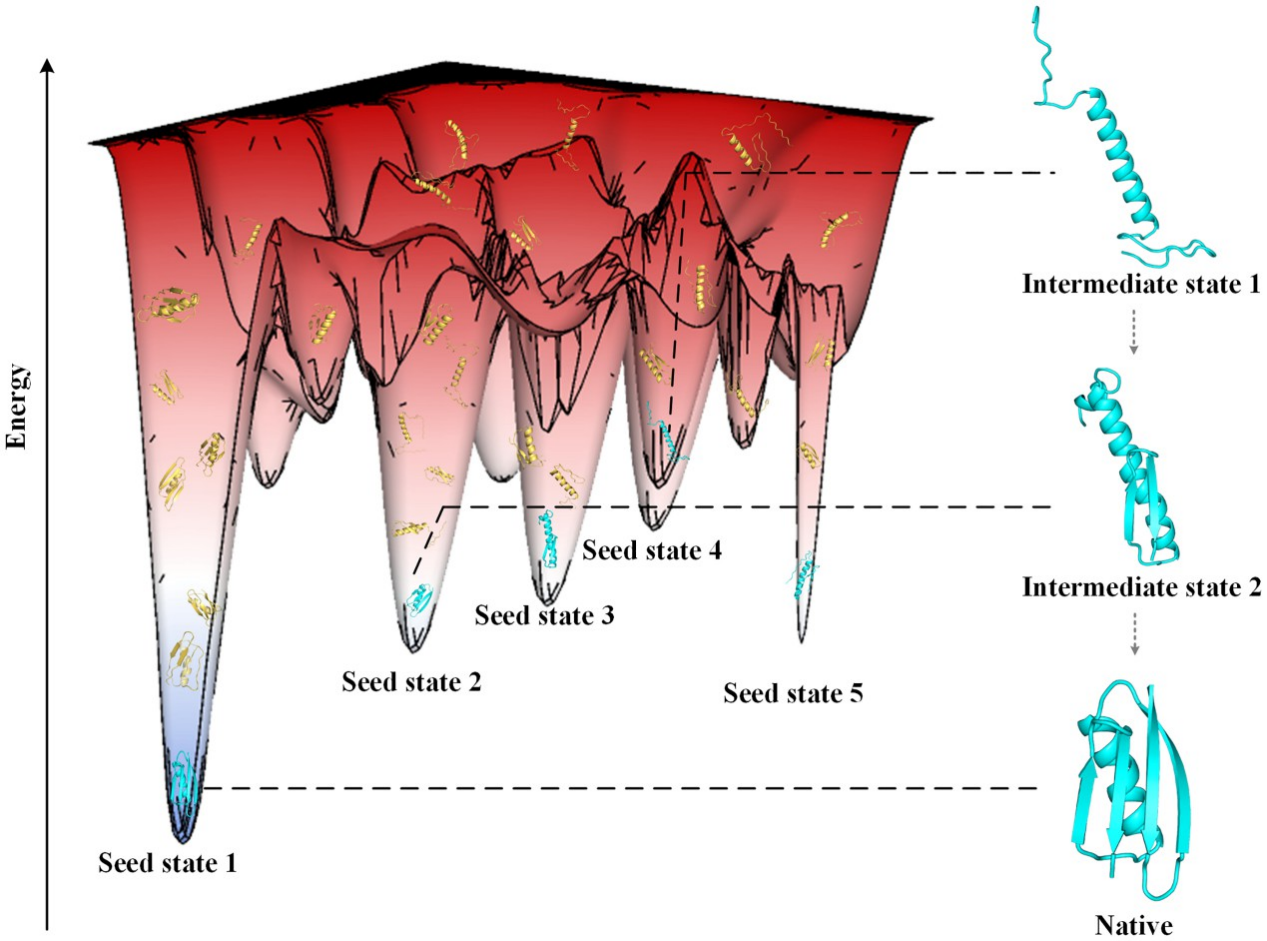
Schematic diagram of folding pathway prediction based on conformational sampling. The yellow structures are sampling states and cyan structures are seed states. The dotted arrows indicate the implicit transition between intermediate states. Sampling states are obtained by the large-scale conformational sampling. The seed states and their transition tendency are inferred by the resampling algorithm.

Protein conformational sampling can provide new ways to explore folding pathways. In this study, we hypothesized that the protein folding information from unfolded state to folded state may be implied in the conformational sampling process in the energy landscape [46,70], and that the maximum probability path of state transition corresponds to the folding pathway. Based on the above assumptions and inspired by hidden Markov model, we predict protein folding pathways by the transition probability between metastable states inferred from sampled conformations. Here, the metastable states located in local energy basins are named as ’Seed states’ (cyan structures, Fig 5), where the states in shallow basin of folding pathway are called ’Intermediate states’. We predicted the folding paths of 34 proteins and analyzed 5 of them in combination with the evaluation metric.

### Datasets

Because the conformational sampling process is greatly affected by the energy force field, we predict the structures of 193 proteins through the basic Rosetta ab initio modeling algorithm and the *ref2015* energy force field. The test set we collected includes 34 proteins. The folding data of 6 proteins are collected from the HDX experimental database of Start2Fold [71].19 proteins are collected from the standardized protein folding database (PFDB) [72], the other 5 proteins come from our collection of related protein folding research papers. The above 30 proteins are included with known protein folding data, and related research papers are in the appendix (Table A in S1 Text). We also collected 4 proteins with no experimental folding information. We take the probability of the maximum transition path after normalization as the confidence level of the prediction result. The folding pathway prediction confidence (Table A in S1 Text) and algorithm running speed (Table D in S1 Text) of these 34 proteins are given.

**Fig 6:**
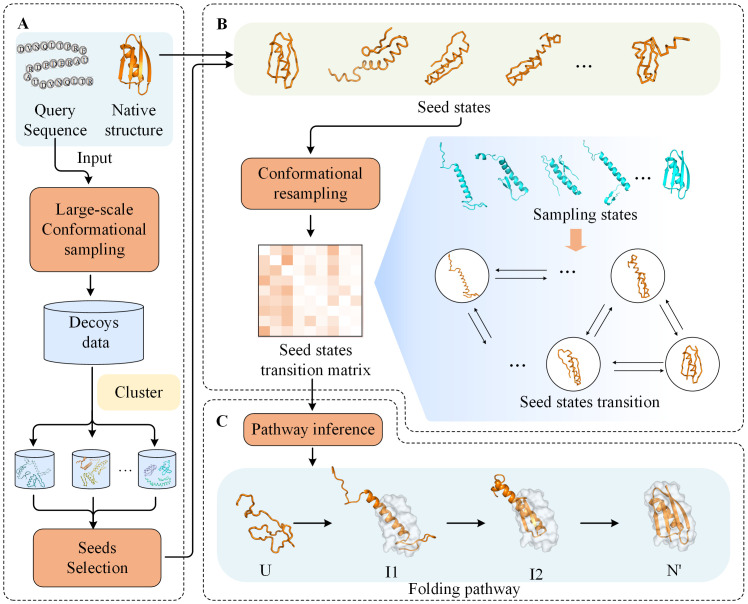
The pipeline of Pathfinder. (A) Seed generation. Sampling of large-scale conformational space by input sequences. Cluster and output the seed states. (B) Transition probability exploration. The seed states in this stage consists of the seeds obtained in stage A and the input native structure. (C) Folding pathway inference. The folding pathway starts from the unfolded state and passes through several intermediate states to a near-native state (N’), which is the closest conformation to native during sampling.

The pipeline of Pathfinder is shown in Fig 6, consists three stages: (A) seed generation, (B) transition probability exploration and (C) folding pathway inference. The input is the query sequence and native structure from PDB (or predicted model by AlphaFold2 if there is no crystal structure in PDB). The output is the predicted protein folding pathway. Guided by the energy function of *ClassicAbinitio* protocol in Rosetta [73,74], fragment assembly-based Metropolis Monte Carlo (MMC) algorithm [75] is used for conformational sampling in the stages (A) and (B). The fragment library is built by the Robetta fragment server (http://old.robetta.org/). In the stage (A), a large-scale conformational sampling algorithm is used to obtain a mass of decoys. The cluster centroid obtained by clustering is selected as the seed state. In the stage (B), based on a modified energy function, conformational resampling is not constrained by local energy traps to explore transition probabilities between seed states. Finally, the protein folding pathways are inferred by transition probabilities using a dynamic programming algorithm in the stage (C).

### Seed generation

We use a large-scale conformational sampling with *G* MMC trajectories to explore energy basins with high repetition rates in folding pathways. Each MMC trajectory generates about 360,000 conformations, where accepted conformations are saved as decoys. There are at least 13000 decoys from all MMC trajectories for cluster. We cluster decoys into centroids using Spicker [76], and every 13,000 decoys are clustered into *S* centroids. Because of redundancy among centroids, we merge centroids with TM-score>*τ* to generate *N* seed states. In particular, the lowest global energy basin is represented in the seed state 1 (illustrated in Fig 5) of the input structure.

### Transition probability exploration

#### Modified energy function

Different from the large-scale sampling, the purpose of resampling algorithms is to explore the transition propensity of the seed states, rather than to simulate de novo protein folding. Because there are masses of energy barriers in the energy landscape, the conformational sampling has the defect that it is difficult to jump out after entering the local energy basin, which leads to low sampling efficiency.

**Fig 7:**
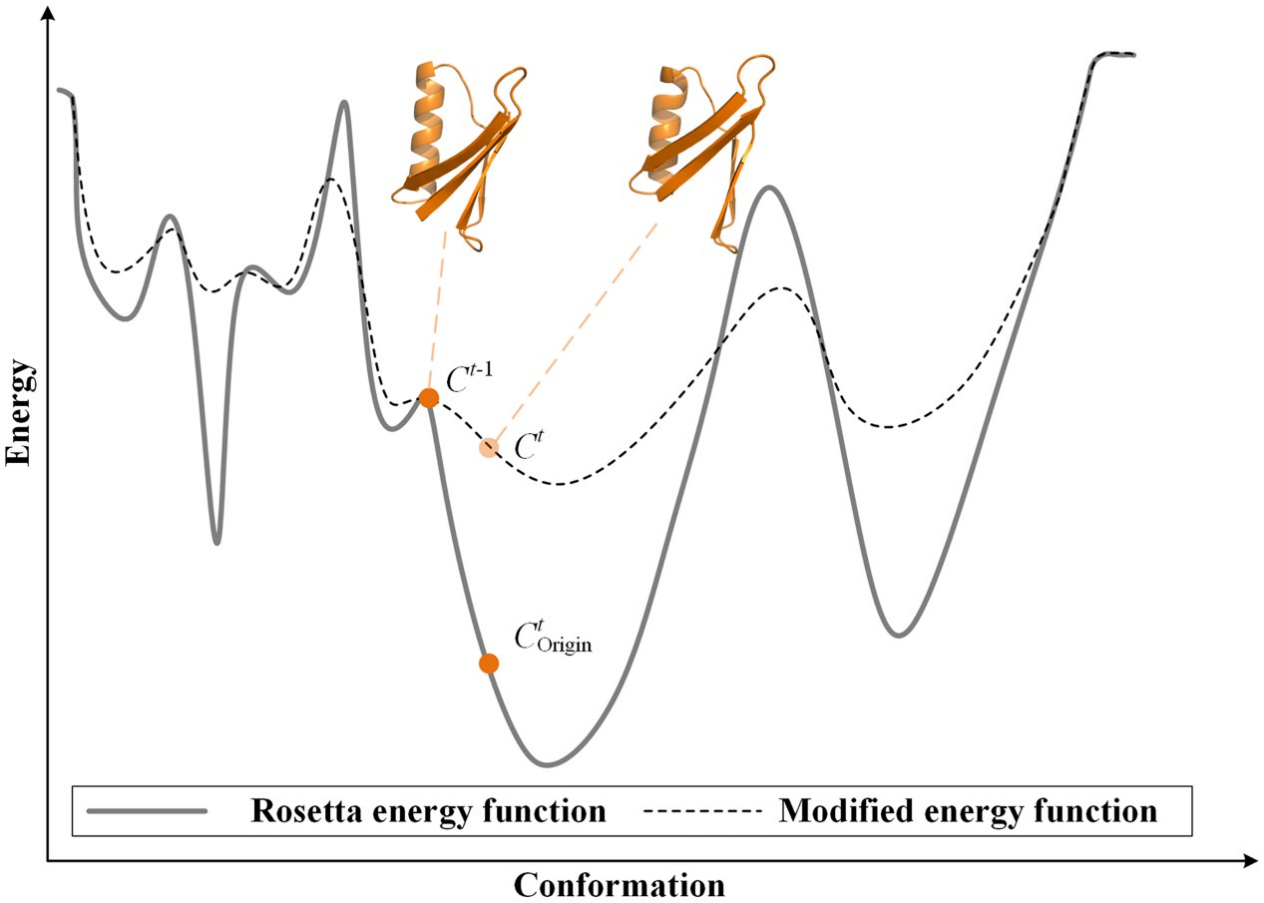
Schematic of the modified energy landscape. After modification, the energy basin is raised and the energy barrier is lowered. And the relatively smooth energy landscape makes transitions between states easy.

Therefore, we construct a modified energy function to facilitate sampling state transitions by raising the energy basin and lowering the energy barrier as illustrate in Fig 7. The *C*^*t*^ is the *t* -th conformation accepted in modified energy landscape, the $C_{\text{origin}}^{t}$ is the original conformation in the unmodified energy landscape, and *C*^*t*−1^ is (*t* − 1) -th conformation. Based on the modified energy function, *C*^*t*^ escapes the local basin more easily than $C_{\text{origin}}^{t}$. The modified energy function *f*(*C*^*t*^) guiding resampling is defined as:

$$f\left(C^{t}\right)=\left\{\begin{array}{ll} E_{r}\left(C^{t}\right), & t=1\\ E_{r}\left(C^{t}\right)+T\left(t\right)arctan(E_{p}\left(C^{t}\right)), & t>1 \end{array}\right.,$$ (3)

where *E*_*r*_(*C*^*t*^) is Rosetta energy function and *E*_*p*_(*C*^*t*^) is an energy function which designed to modify the original energy function. *T*(*t*) is reduced as the number of samples increases to offset the large energy gap between the unfolded state and the folded state.

$$T\left(t\right)=\frac{L}{\left(t+\mu \right)},$$ (4)

where *L* is the length of the protein, the *μ* is the initial value to avoid excessive energy at the beginning of sampling. The energy function *E*_*p*_(*C*^*t*^) is designed as:

$$E_{p}\left(C^{t}\right)=E_{p}\left(C^{t-1}\right)+\frac{\left(E_{r}\left(C^{t-1}\right)-E_{r}\left(C^{t}\right)\right)}{\sqrt{S\left(C^{t},C^{t-1}\right)}},\;t>1,$$ (5)

where *E*_*p*_(*C*^*t*−1^) is the previously accumulated energy function to maintain enough energy to rush out of the energy basin. *S*(*C*^*t*^, *C*^*t*−1^) is the dihedral angles difference between *C*^*t*^ and *C*^*t*−1^, designed as:

$$S\left(C^{t},C^{t-1}\right)=\frac{1}{3L}\sum_{i=1}^{L}{\left(\varphi_{i}^{t}-\varphi_{i}^{t-1}\right)}^{2}+{\left(\phi_{i}^{t}-\phi_{i}^{t-1}\right)}^{2}+{\left(\omega_{i}^{t}-\omega_{i}^{t-1}\right)}^{2},$$ (6)

where $\varphi_{i}^{t},\;\phi_{i}^{t}$ and $\omega_{i}^{t}$ are the dihedral angles of *i* residue of *t* -th conformation. The modified energy function was compared with the benchmark conformational sampling algorithm (Fig E in S1 Text). The results show that the modified energy function can explore wider energy basins and guide the conformational sampling process more quickly.

#### Seed states transition generation

In the funnel model, high-energy barriers exist around local energy basins, and random jumps in sampling points between basins contain transition information [35]. The resampling algorithm locates energy basins for sampling in conformational space based on sampling states and DMscore cutoff η. DMscore [50] is a structural similarity metric we previously developed, focusing on secondary structure to determine the extent of local energy basins. Based on the modified energy function, the resampling algorithm utilizes conformational sampling to obtain potential seed state transition probabilities. Inspired by hidden Markov models, the observation state is a representation of the hidden unknown state. The state maximum probability path can be obtained by constructing the model and the observation state. However, state transition path inference needs to obtain a continuous sequence of observation states and cannot be directly used in random image sampling methods. Therefore, we generate a mass of sampling states to obtain state transitions by comparing their structural similarity with the seed states. MMC trajectories are different from the continuous trajectories of molecular dynamics simulations, including random conformational structure transitions. We define the state transition frequency matrix as ***B*** = {*b*_*ij*_}, where *b*_*ij*_ is obtain by resampling algorithm. We consider the sampled trajectory to enter this state region when the DMscore between the sampling conformation and seed *i* is greater than *ξ*. At the same time, we designed the unidirectionality of the transformation, that is, when the transmission from *i* to *j* is the reverse of the previous transmission, the transmission frequency is not calculated, which helps to reduce the background noise generated by random sampling. The transition frequency of the seed states is calculated by resampling. We further process the ***B*** to get the transition probability matrix ***A*** = {*a*_*ij*_}, where $a_{ij}=\frac{b_{ij}}{\sum_{j}^{N}b_{ij}}$.

### Folding pathway inference

Based on the seed states transition probability matrix ***A***, protein folding pathways are inferred using a dynamic programming algorithm. The folding pathways are represented in the order of seed states, which are inferred from the transition probability matrix. Based on the *N* seed states obtained above, the optimal path is defined as $I^{*}=(i_{1}^{*},i_{2}^{*},\ldots ,i_{n}^{*},\ldots ,i_{N}^{*})$, where $i_{n}^{*}$ is the optimal path from $i_{1}^{*}$ to $i_{n}^{*}$. The $i_{n}^{*}=\psi_{n}(i)$ is defined as:

$$\begin{array}{ll} \psi_{n}\left(i\right)=arg\;\underset{1\leq j\leq N}{max}\;p_{n}\left(i\right), & i=1,2,\ldots ,N, \end{array}$$ (7)

$$\begin{array}{ll} p_{n}\left(i\right)=\underset{1\leq j\leq N}{max}\;p_{n-1}\left(j\right)a_{ji}, & i=1,2,\ldots ,N, \end{array}$$ (8)

where *p*_*n*_(*i*) is the maximum transition probability to $i_{n}^{*}$, and *a*_*ji*_ is the transition probability from seed *i* to seed *j*. Because of the imperfection of the Rosetta force field, it is difficult to sample protein conformations to the native state. Therefore, the known native structure is used as the end point of the folding (*ψ*_1_(*i*) = 1), and the folding pathway I* is reversely inferred.

## Supporting information

S1 Text Supplementary tables and figures. **Fig A. 11 validated protein folding pathways. Fig B. 13 protein folding pathways to be verified. Fig C. 6 defective protein folding pathways. Fig D. Two cases showing limitation of contact order. (a)** is the contact order and intermediate state line diagram of 5l8i protein. **(b)** is the intermediate state structure of 5l8i protein. **(c)** is the line graph of contact order and intermediate state of 1opa protein. **(d)** is the intermediate state structure of 1opa protein. It can be found that the contact order of intermediate state 1 is relatively high because there is a cavity in the middle of the structure in the native state. In the metastable structure, the contact order of the intermediate state 1 is higher than that of the intermediate state 2, which is about to form the cavity, because the cavity has not yet been formed. **Fig E. The spline connection graph of the normalized residue contact order. (a)** is a distance map of the intermediate states of the GB1 protein. **(b)** is the residue contact order diagram of the intermediate state of GB1 protein, where orange represents the intermediate state and gray represents the native structure. **(c)** The ratio of the residue contact order between the intermediate state and the native structure is represented in color on the structure. The more similar the residue contact order in the intermediate state is to the natural structure, the higher the ratio, and the more it tends to blue. Comparing the residue contact order with the native structure, the folding degree of the intermediate state and the order of appearance of the secondary structure can be further analyzed. Furthermore, the residue contact order information can be represented by a three-dimensional structure to better observe the folding nucleus. **Fig F. LB1 sampling process diagram.** A total of 10125 accepted process points were generated during one conformational sampling process of LB1. We gave the first 3000 sampling data and analyzed the first 30 conformations. And present part of the conformation. **Fig G. Predicted folding pathway of NuG2.** The probability of the I1, I2 and N’ pathway is 0.67 and the probability of the I1, I3 and N’ pathway is 0.32. **Fig H. Folding pathway prediction results of helical proteins.** Pathfinder’s predictions for proteins containing only α-helices may be biased. The intermediate state predicted by Pathfinder may complete the assembly of the α-helix of each link in the early stage of folding, so that the sampling of the late stage is more about the sampling of the loop region. However, the experimental results shown that the helix at both ends of the 1yyj protein is folded to be stable at the late stage of folding. **Fig I. Line graph of sampling times and energy. (a)** is the sampling trajectory of 1e0m protein, and **(b)** is the sampling trajectory of 1opa. Among them, the protein energy of 1e0m has almost no drop, and 1opa is more in line with the normal conformational sampling process. **Fig J. Prediction of defective protein folding pathway analysis.** We analyzed the cause of insufficient folding pathways for other proteins (PDB ID: 1hdn, 1tp3, 1ten, 1ubq, 3chy). Using *score_jd2* protocol to calculate the intermediate state of these proteins based on the *ref2015* scoring function. We further use *fastrelax* to relax the intermediate states. The processed intermediate state also uses the *score_jd2* protocol to calculate the energy item, which is shown in the fourth column of the figure. The results show that the intermediate states of 1ten and 1ubq proteins follow the sampling process of energy decline in the *ref2015* energy force field, which indicates that the energy force field is biased in sampling the intermediate states of these two proteins. **Fig K. Sampling Process Analysis Diagram. (a)** is the energy and RMSD scatterplot of the conformations of the sampling process. Red dots are the conformations of the sampling process under the modified energy force field. Black dots are the benchmark conformations of Rosetta’s conformational sampling process. **(b)** is the trajectory of energy variation with the number of samples. Red is the sampling trace of the modified energy force field, and black is the sampling trace of the baseline Rosetta. **Table A. The test set of Pathfinder. Table B. The parameter of Pathfinder. Table C. Performance improvement table of modified energy function (MEF). Table D. Table Running Speed of Pathfinder on dataset**.
(DOCX)
