# Supplementary material for: Pathfinder: Protein folding pathway prediction based on conformational sampling
Source: PLoS Comput Biol. 2023 Sep 11;19(9):e1011438. doi: 10.1371/journal.pcbi.1011438 (PMC10513300; doi:10.1371/journal.pcbi.1011438)
Supplement: S1 Text — Fig A. 11 validated protein folding pathways. Fig B. 13 protein folding pathways to be verified. Fig C. 6 defective protein folding pathways. Fig D. Two cases showing limitation of contact order. (a) is the contact order and intermediate state line diagram of 5l8i protein. (b) is the intermediate state structure of 5l8i protein. (c) is the line graph of contact order and intermediate state of 1opa protein. (d) is the intermediate state structure of 1opa protein. It can be found that the contact order of intermediate state 1 is relatively high because there is a cavity in the middle of the structure in the native state. In the metastable structure, the contact order of the intermediate state 1 is higher than that of the intermediate state 2, which is about to form the cavity, because the cavity has not yet been formed. Fig E. The spline connection graph of the normalized residue contact order. (a) is a distance map of the intermediate states of the GB1 protein. (b) is the residue contact order diagram of the intermediate state of GB1 protein, where orange represents the intermediate state and gray represents the native structure. (c) The ratio of the residue contact order between the intermediate state and the native structure is represented in color on the structure. The more similar the residue contact order in the intermediate state is to the natural structure, the higher the ratio, and the more it tends to blue. Comparing the residue contact order with the native structure, the folding degree of the intermediate state and the order of appearance of the secondary structure can be further analyzed. Furthermore, the residue contact order information can be represented by a three-dimensional structure to better observe the folding nucleus. Fig F. LB1 sampling process diagram. A total of 10125 accepted process points were generated during one conformational sampling process of LB1. We gave the first 3000 sampling data and analyzed the first 30 conformations. And prese [file pcbi.1011438.s001.docx]

**Pathfinder:** **protein folding pathway prediction based on conformational sampling**

Supporting Information

Zhaohong Huang^1¶^, Xinyue Cui^1¶^, Yuhao Xia^1^, Kailong Zhao^1^ and Guijun Zhang^1∗^

^1^College of Information Engineering, Zhejiang University of Technology, Hangzhou, China.

zgj@zjut.edu.cn.

^¶^These authors contributed equally to this work.


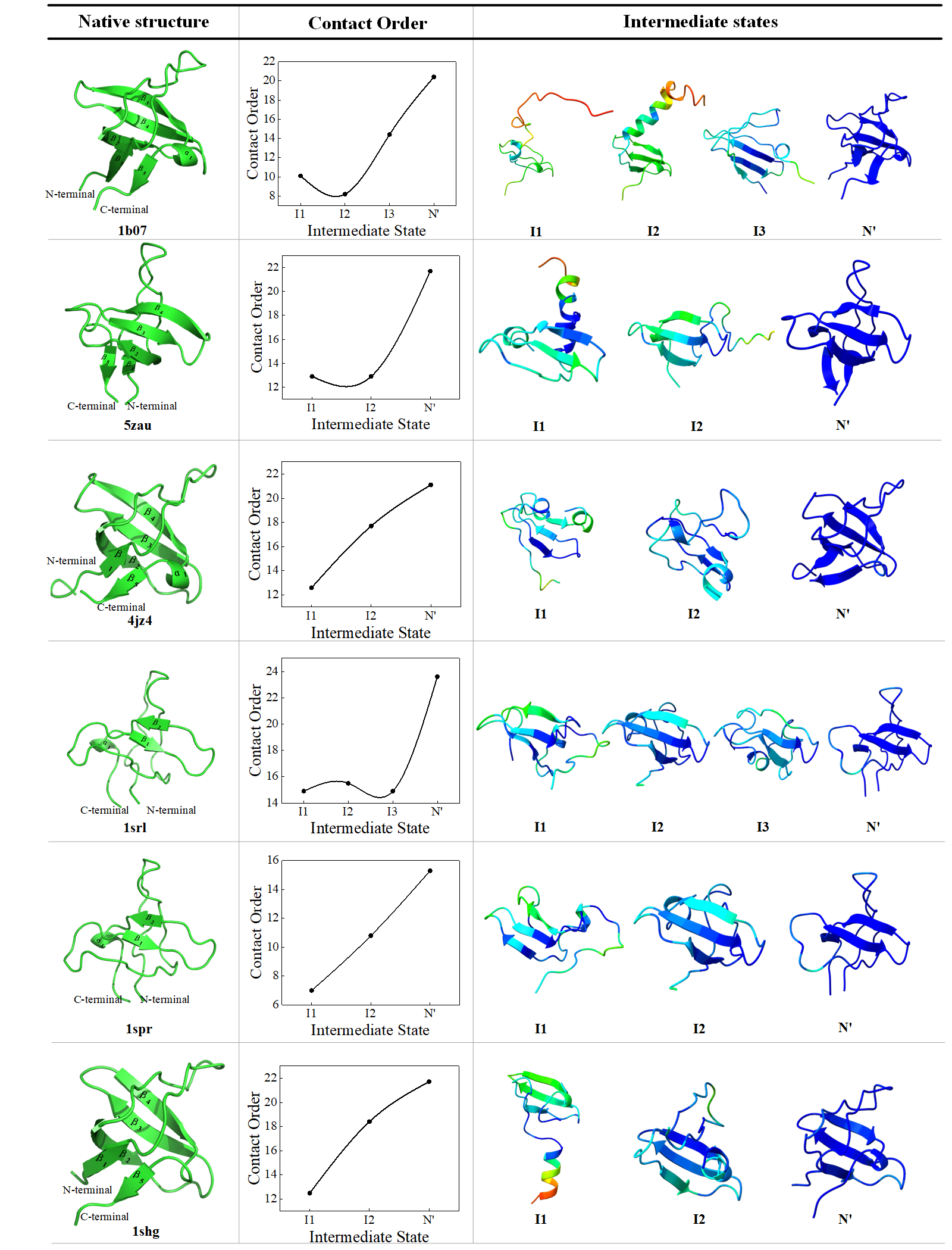


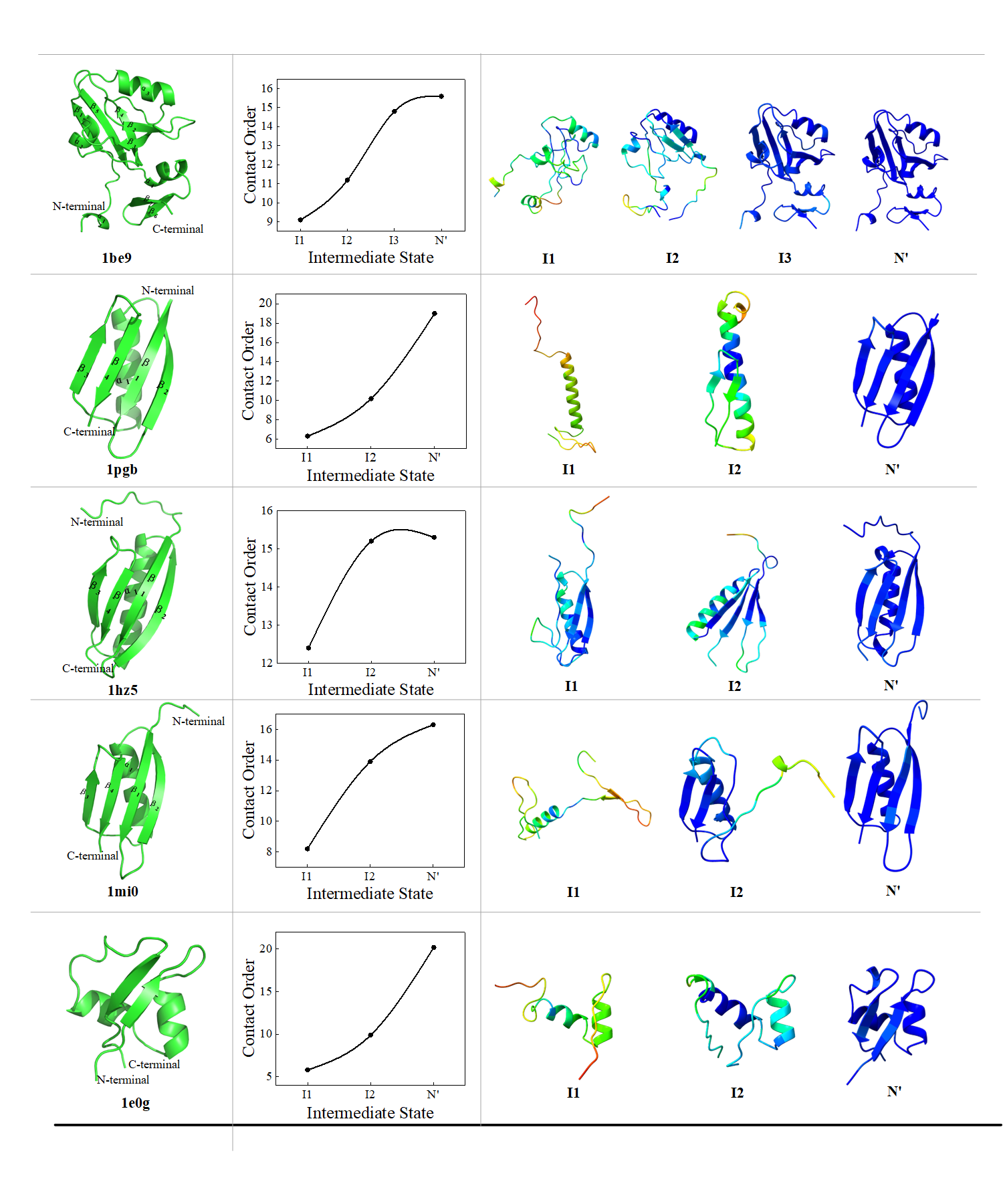


**Fig A.** **11 validated protein folding pathways.**


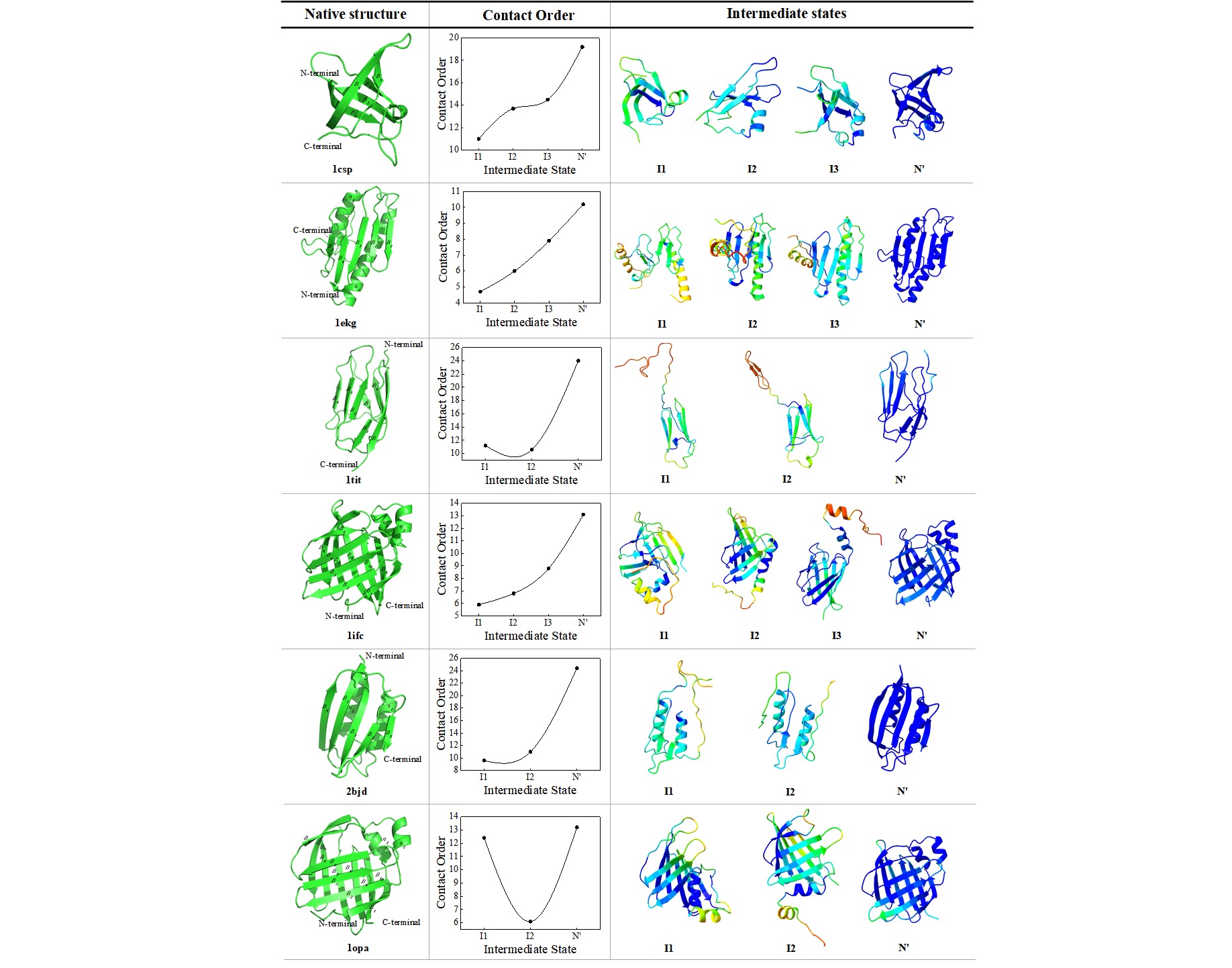


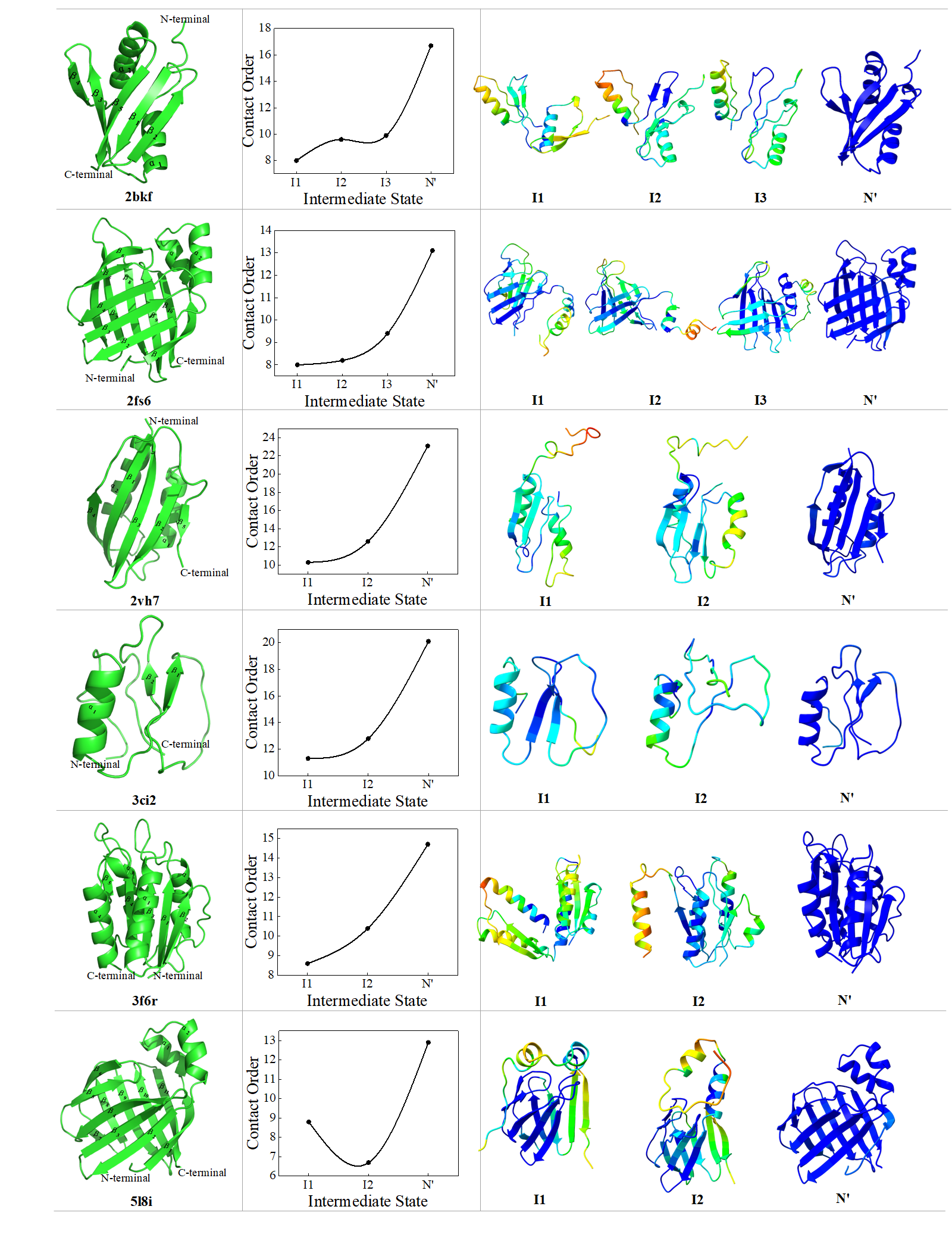


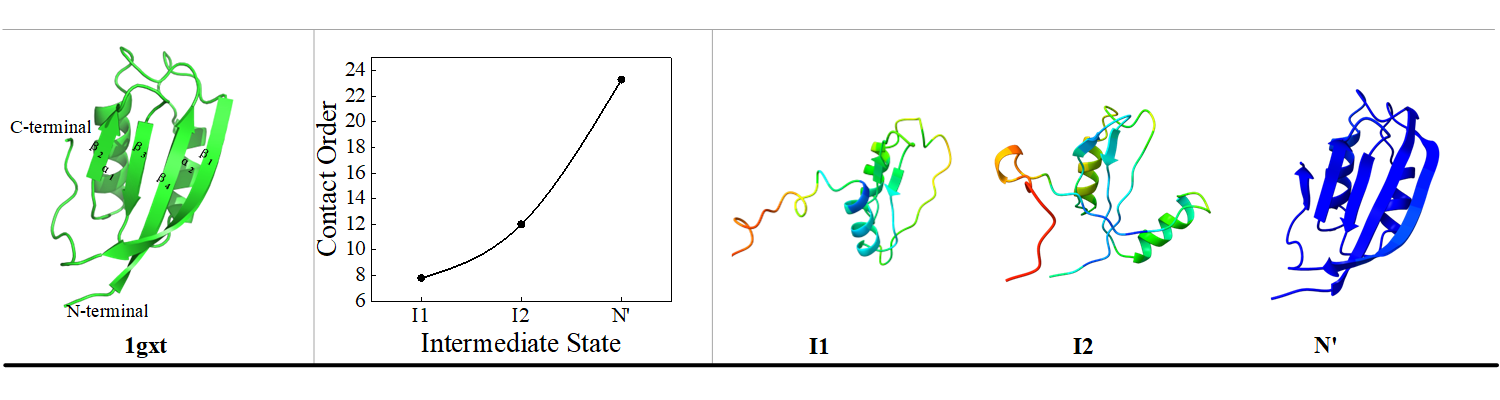


**Fig B.** **13 protein folding pathways to be verified.**


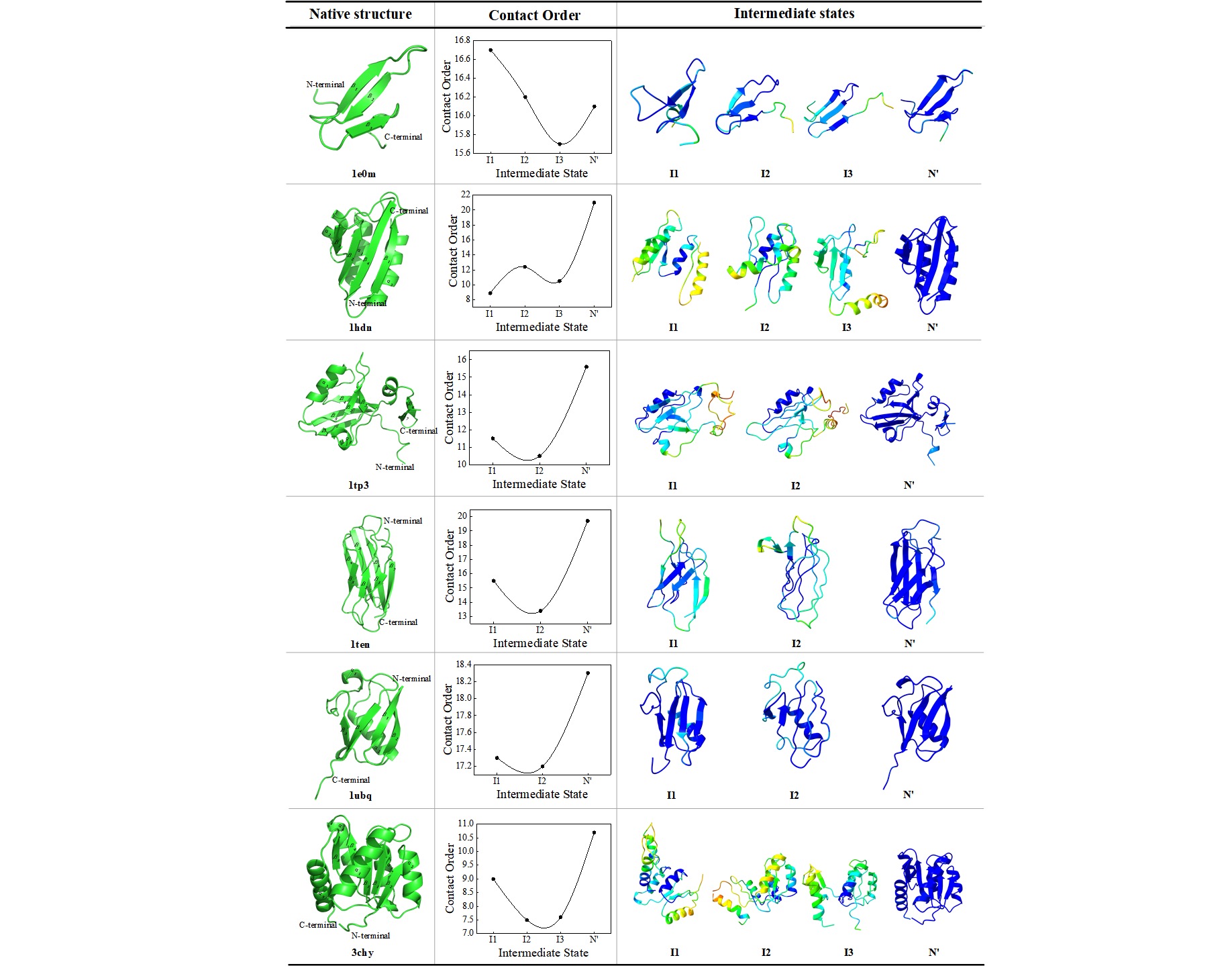


**Fig C.** **6 defective protein folding pathways.**


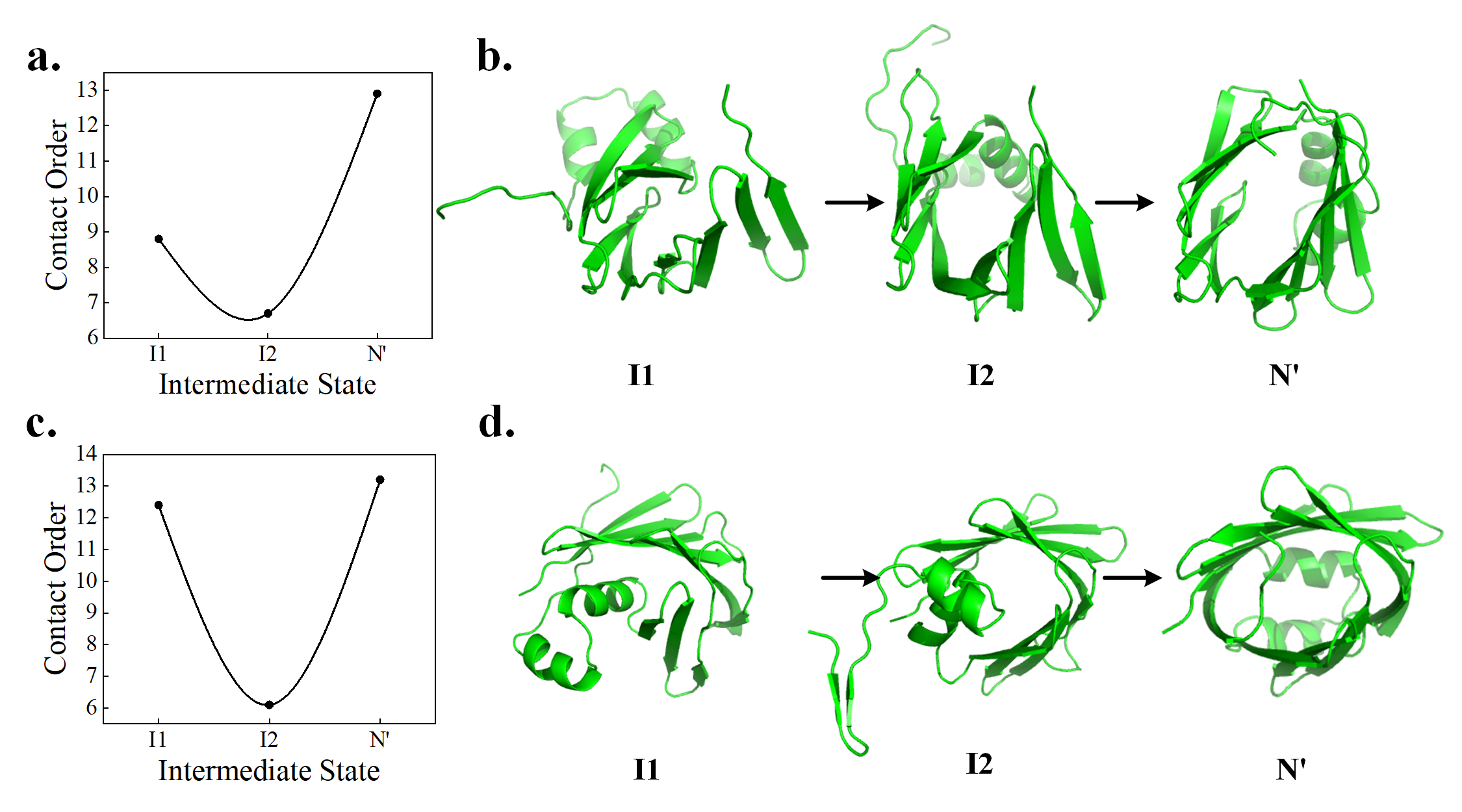


**Fig D.** **Two cases showing limitation of contact order.**

(a) is the contact order and intermediate state line diagram of 5l8i protein. (b) is the intermediate state structure of 5l8i protein. (c) is the line graph of contact order and intermediate state of 1opa protein. (d) is the intermediate state structure of 1opa protein. It can be found that the contact order of intermediate state 1 is relatively high because there is a cavity in the middle of the structure in the native state. In the metastable structure, the contact order of the intermediate state 1 is higher than that of the intermediate state 2, which is about to form the cavity, because the cavity has not yet been formed.


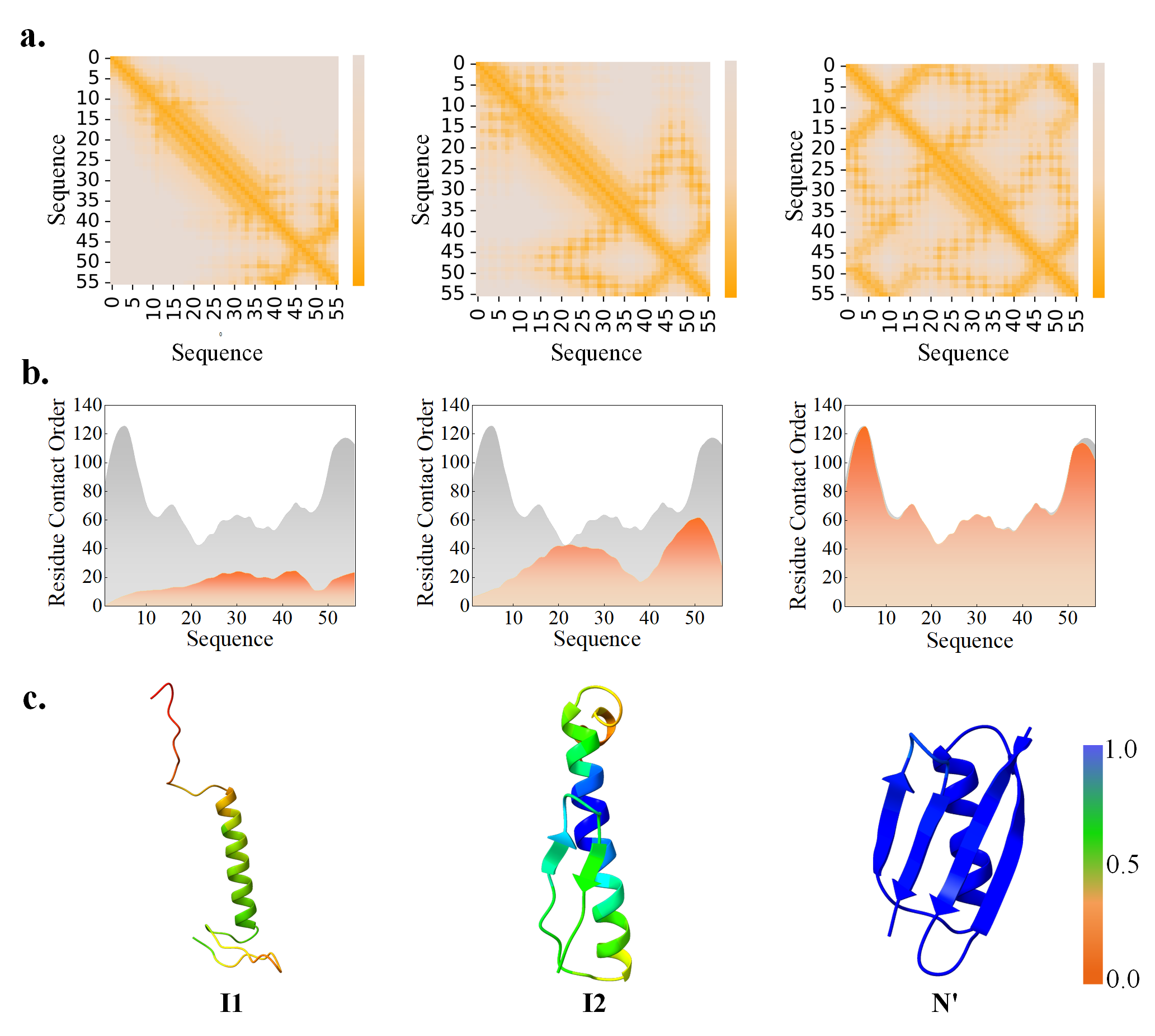


**Fig E.** **The spline connection graph of the normalized residue contact order.**

**(a)** is a distance map of the intermediate states of the GB1 protein. **(b)** is the residue contact order diagram of the intermediate state of GB1 protein, where orange represents the intermediate state and gray represents the native structure. **(c)** The ratio of the residue contact order between the intermediate state and the native structure is represented in color on the structure. The more similar the residue contact order in the intermediate state is to the natural structure, the higher the ratio, and the more it tends to blue. Comparing the residue contact order with the native structure, the folding degree of the intermediate state and the order of appearance of the secondary structure can be further analyzed. Furthermore, the residue contact order information can be represented by a three-dimensional structure to better observe the folding nucleus.


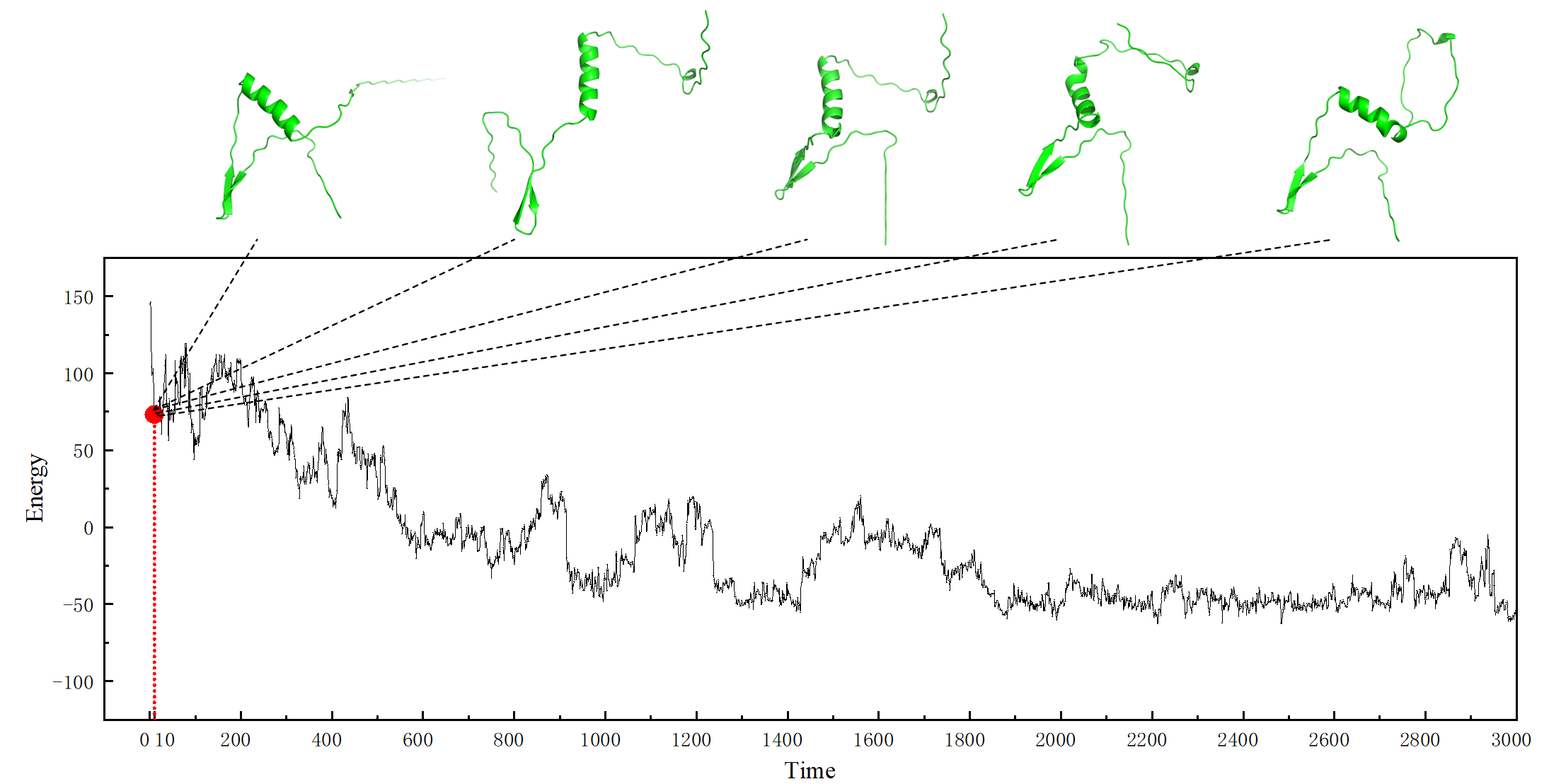


**Fig F. LB1 sampling process diagram.**

A total of 10125 accepted process points were generated during one conformational sampling process of LB1. We gave the first 3000 sampling data and analyzed the first 30 conformations. And present part of the conformation.


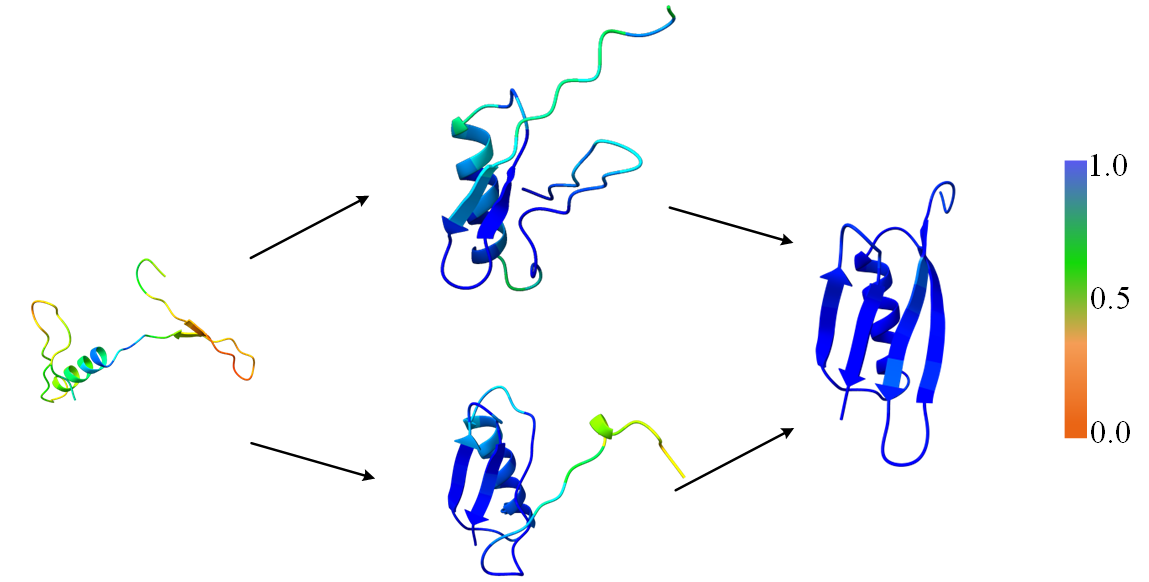


**Fig G. Predicted folding pathway of NuG2.**

The probability of the I1, I2 and N' pathway is 0.67 and the probability of the I1, I3 and N' pathway is 0.32.


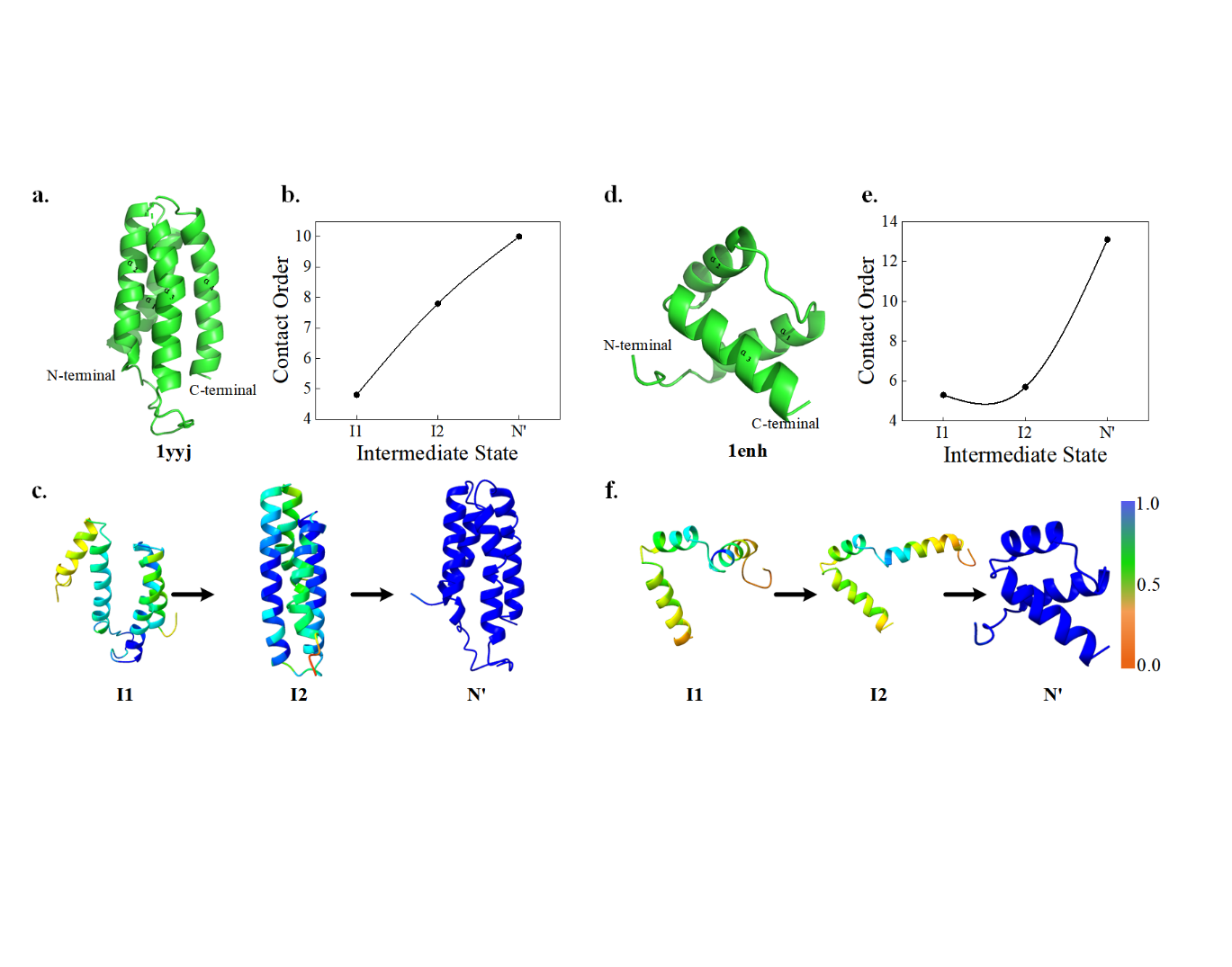


**Fig H.** **Folding pathway prediction results of helical proteins.**

Pathfinder's predictions for proteins containing only α-helices may be biased. The intermediate state predicted by Pathfinder may complete the assembly of the α-helix of each link in the early stage of folding, so that the sampling of the late stage is more about the sampling of the loop region. However, the experimental results shown that the helix at both ends of the 1yyj protein is folded to be stable at the late stage of folding.


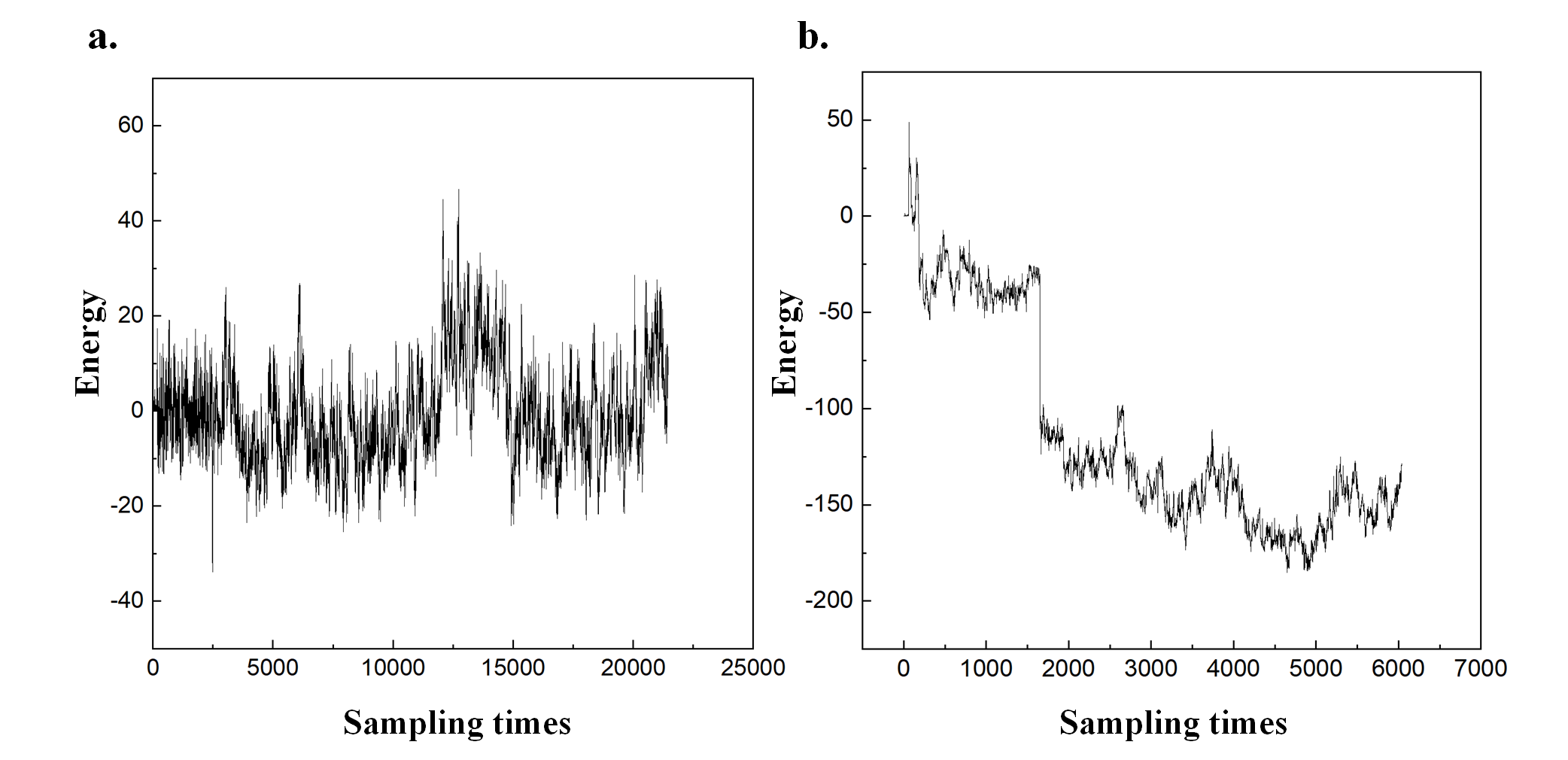


**Fig I.**  **Line graph of sampling times and energy.**

**(a)** is the sampling trajectory of 1e0m protein, and **(b)** is the sampling trajectory of 1opa. Among them, the protein energy of 1e0m has almost no drop, and 1opa is more in line with the normal conformational sampling process.


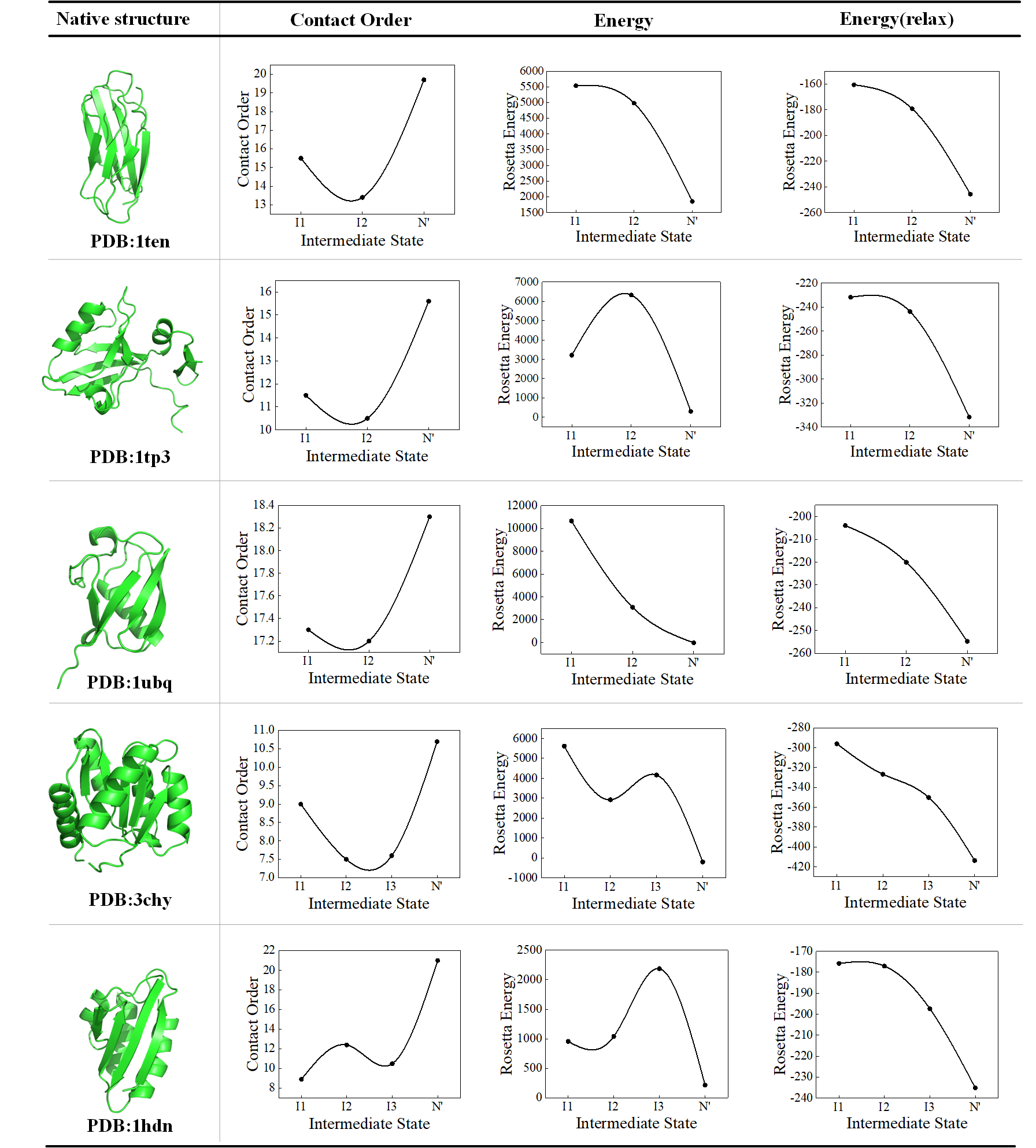


**Fig J.** **Prediction of defective protein folding pathway analysis.**

We analyzed the cause of insufficient folding pathways for other proteins (PDB ID: 1hdn, 1tp3, 1ten, 1ubq, 3chy). Using *score_jd2* protocol to calculate the intermediate state of these proteins based on the *ref2015* scoring function. We further use *fastrelax* to relax the intermediate states. The processed intermediate state also uses the *score_jd2* protocol to calculate the energy item, which is shown in the fourth column of the figure. The results show that the intermediate states of 1ten and 1ubq proteins follow the sampling process of energy decline in the *ref2015* energy force field, which indicates that the energy force field is biased in sampling the intermediate states of these two proteins.


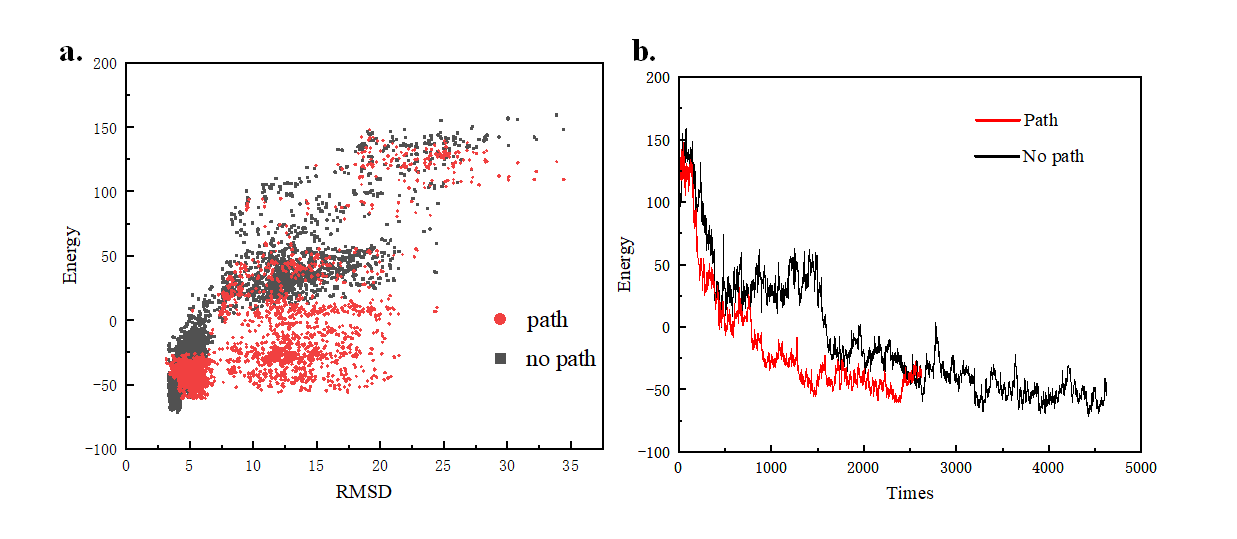


**Fig K.** **Sampling Process Analysis Diagram.**

**(a)** is the energy and RMSD scatterplot of the conformations of the sampling process. Red dots are the conformations of the sampling process under the modified energy force field. Black dots are the benchmark conformations of Rosetta's conformational sampling process. **(b)** is the trajectory of energy variation with the number of samples. Red is the sampling trace of the modified energy force field, and black is the sampling trace of the baseline Rosetta.

**Table A**. **The test set of Pathfinder**

| PDB | Confidence | Length | Name | DOI |
| --- | --- | --- | --- | --- |
| 1b07 | 0.95 | 59 | crk sh3 domain | 10.1126/science.1214203 |
| 1be9 | 0.61 | 115 | third pdz domain from the synaptic protein psd-95 | 10.1016/s0092-8674(00)81307-0 |
| 1csp | 0.31 | 67 | cspb: a universal nucleic-acid binding domain | 10.1038/nsb0398-229 |
| 1e0g | 0.84 | 48 | lysm domain from e.coli mltd | 10.1016/j.jmb.2008.05.020 |
| 1e0m | 0.51 | 37 | prototype ww domain | 10.1073/pnas.221467198 |
| 1ekg | 0.46 | 119 | mature human frataxin | 10.1038/srep20782 |
| 1gxt | 0.62 | 88 | hydrogenase maturation protein | 10.1016/S0022-2836(03)00627-2 |
| 1hdn | 0.86 | 85 | histidine-containing phosphocarrier protein hpr | 10.1021/bi9717946 |
| 1hz5 | 0.82 | 72 | b1 domain of protein l | 10.1038/89638 |
| 1ifc | 0.61 | 131 | recombinant rat intestinal fatty acid-binding apoprotein | 10.1021/bi012042l |
| 1mi0 | 0.66 | 61 | the redesigned protein g variant nug2 | 10.1038/89638 |
| 1opa | 0.96 | 133 | holo-and apo-cellular retinol binding protein ii | 10.1002/(SICI)1097-0134(19981001)33:1<107::AID-PROT10>3.0.CO;2-P |
| 1pgb | 0.82 | 56 | b1 immunoglobulin-binding domain of streptoccocal protein g | 10.1038/13311 |
| 1shg | 0.94 | 57 | sh3 domain | 10.1110/ps.041205405 |
| 1spr | 0.74 | 103 | src sh2 domain | 10.1110/ps.041205405 |
| 1srl | 0.98 | 56 | src sh3 domain | 10.1110/ps.041205405 |
| 1ten | 0.40 | 89 | a fibronectin type iii domain from tenascin phased | 10.1006/jmbi.2000.3517 |
| 1tit | 0.30 | 89 | titin, ig repeat 27, | 10.1016/s0969-2126(01)00596-2 |
| 1tp3 | 0.94 | 115 | pdz3 domain of psd-95 protein | 10.1073/pnas.0804774105 |
| 1ubq | 0.40 | 76 | ubiquitin | 10.1021/cr040430y |
| 2bjd | 0.51 | 90 | sulfolobus solfataricus acylphosphatase. triclinic space group | 10.1021/bi030238a |
| 2bkf | 0.44 | 86 | pb1 domain of nbr1 | 10.1021/bi1016793 |
| 2fs6 | 0.71 | 136 | apo-cellular retinoic acid binding protein type ii | 10.1002/prot.1040 |
| 2vh7 | 0.82 | 94 | human common-type acylphosphatase | 10.1021/bi9822630 |
| 3chy | 0.64 | 128 | escherichia coli chey | 10.1021/bi00185a025 |
| 3ci2 | 0.56 | 63 | barley serine proteinase inhibitor 2 | 10.1110/ps.041205405 |
| 3f6r | 0.34 | 147 | desulfovibrio desulfuricans (atcc 29577) oxidized flavodoxin | 10.1016/j.jmb.2009.11.008 |
| 4jz4 | 0.63 | 60 | chicken c-src-sh3 domain | 10.1126/science.1214203 |
| 5l8i | 0.58 | 125 | human fabp6 apo-protein | 10.1002/prot.22286 |
| 5zau | 0.49 | 57 | human fyn sh3 and monobody binder | 10.1126/science.1214203 |
| 2qzj | 0.98 | 136 | Two-component response regulator from Clostridium difficile | - |
| 3ilh | 0.46 | 146 | Two component response regulator from Cytophaga hutchinsonii | - |
| 4qpj | 0.45 | 243 | Phosphotransferase in Complex with a Receiver Domain | - |
| 6swl | 0.73 | 133 | response regulator from Geobacillus stearothermophilus | - |

*Among the 34 proteins, 30 have related literatures describing their folding pathways, and the remaining 4 proteins have no related protein folding pathways.

**Table B**. **The parameter of Pathfinder**

| Parameter | Default Value |
| --- | --- |
| *G* | 10 |
| *S* | 10 |
| *τ* | 0.55 |
| *μ* | 1000 |
| *η* | 0.5 |
| *ξ* | 0.7 |

**Table C.** **Performance improvement table of modified energy function (MEF)**

| Methods | TM-score | Sample_time | Running_time(min) |
| --- | --- | --- | --- |
| MEF | **0.4258** | **23813** | **185** |
| Rosetta | 0.4254 | 27227 | 193 |

**Table D.** **Running Speed of Pathfinder on dataset**

| NO. | PDB | Length | Stage1 | Stage2 | Stage1+Stage2 | All |
| --- | --- | --- | --- | --- | --- | --- |
| 1 | 1b07 | 59 | 1:01:30 | 3:46:44 | 4:15:21 | 4:48:14 |
| 2 | 1be9 | 115 | 4:26:30 | 11:11:23 | 13:24:44 | 15:37:53 |
| 3 | 1csp | 67 | 1:35:02 | 4:00:46 | 6:26:26 | 7:23:09 |
| 4 | 1e0g | 48 | 0:43:10 | 2:10:57 | 3:19:43 | 4:43:56 |
| 5 | 1e0m | 37 | 0:40:24 | 9:10:34 | 2:22:35 | 2:51:21 |
| 6 | 1ekg | 119 | 1:50:48 | 9:58:25 | 10:53:39 | 11:01:22 |
| 7 | 1gxt | 88 | 1:20:50 | 6:41:27 | 8:02:17 | 9:15:08 |
| 8 | 1hdn | 85 | 0:35:01 | 4:13:11 | 4:48:12 | 5:22:44 |
| 9 | 1hz5 | 72 | 0:55:33 | 4:53:23 | 5:48:56 | 6:38:08 |
| 10 | 1ifc | 131 | 2:46:56 | 9:11:44 | 11:58:40 | 13:04:16 |
| 11 | 1mi0 | 61 | 0:30:45 | 2:23:45 | 2:54:30 | 3:14:41 |
| 12 | 1opa | 133 | 3:12:23 | 9:10:27 | 12:22:50 | 14:07:55 |
| 13 | 1pgb | 56 | 0:53:48 | 1:25:27 | 2:19:15 | 2:42:34 |
| 14 | 1shg | 57 | 0:29:48 | 1:40:49 | 2:10:37 | 2:29:37 |
| 15 | 1spr | 103 | 0:55:34 | 9:13:02 | 10:08:36 | 10:49:25 |
| 16 | 1srl | 56 | 0:26:57 | 2:15:17 | 2:42:14 | 2:50:31 |
| 17 | 1ten | 89 | 2:09:30 | 3:44:36 | 5:54:06 | 5:58:59 |
| 18 | 1tit | 89 | 1:32:25 | 4:25:18 | 5:57:43 | 6:17:26 |
| 19 | 1tp3 | 115 | 2:10:01 | 10:51:02 | 13:01:03 | 14:58:14 |
| 20 | 1ubq | 76 | 0:57:21 | 3:47:39 | 4:45:00 | 4:59:36 |
| 21 | 2bjd | 90 | 1:45:43 | 6:06:30 | 7:52:13 | 8:49:36 |
| 22 | 2bkf | 86 | 1:05:37 | 5:05:17 | 6:10:54 | 6:51:07 |
| 23 | 2fs6 | 136 | 2:56:40 | 10:37:31 | 13:34:11 | 15:29:22 |
| 24 | 2qzj | 94 | 1:54:40 | 3:48:20 | 5:43:00 | 6:31:56 |
| 25 | 2vh7 | 128 | 1:52:55 | 6:54:08 | 8:47:03 | 9:32:24 |
| 26 | 3chy | 63 | 2:23:54 | 3:49:37 | 6:13:31 | 6:51:05 |
| 27 | 3ci2 | 147 | 0:25:19 | 4:56:46 | 5:22:05 | 7:30:36 |
| 28 | 3f6r | 60 | 3:01:17 | 13:48:45 | 16:50:02 | 19:02:22 |
| 29 | 3ilh | 125 | 2:34:15 | 7:37:46 | 10:12:01 | 14:38:01 |
| 30 | 4jz4 | 57 | 0:30:34 | 2:57:31 | 3:28:05 | 5:11:22 |
| 31 | 4qpj | 136 | 2:08:39 | 3:36:26 | 5:45:05 | 8:14:27 |
| 32 | 5l8i | 146 | 3:05:22 | 8:52:57 | 11:58:19 | 16:41:43 |
| 33 | 5zau | 243 | 0:25:28 | 1:55:11 | 2:20:39 | 6:14:37 |
| 34 | 6swl | 133 | 1:29:49 | 5:35:35 | 7:05:24 | 11:35:16 |
| **avg** | - | 97 | 1:36:54 | 5:52:53 | 7:12:19 | 8:36:09 |
